# Molecular Evolution of the Neuropeptide S Receptor

**DOI:** 10.1371/journal.pone.0034046

**Published:** 2012-03-30

**Authors:** Thejkiran Pitti, Narayanan Manoj

**Affiliations:** Department of Biotechnology, Indian Institute of Technology Madras, Chennai, India; University of South Florida College of Medicine, United States of America

## Abstract

The neuropeptide S receptor (NPSR) is a recently deorphanized member of the G protein-coupled receptor (GPCR) superfamily and is activated by the neuropeptide S (NPS). NPSR and NPS are widely expressed in central nervous system and are known to have crucial roles in asthma pathogenesis, locomotor activity, wakefulness, anxiety and food intake. The NPS-NPSR system was previously thought to have first evolved in the tetrapods. Here we examine the origin and the molecular evolution of the NPSR using *in-silico* comparative analyses and document the molecular basis of divergence of the NPSR from its closest vertebrate paralogs. In this study, NPSR-like sequences have been identified in a hemichordate and a cephalochordate, suggesting an earlier emergence of a NPSR-like sequence in the metazoan lineage. Phylogenetic analyses revealed that the NPSR is most closely related to the invertebrate cardioacceleratory peptide receptor (CCAPR) and the group of vasopressin-like receptors. Gene structure features were congruent with the phylogenetic clustering and supported the orthology of NPSR to the invertebrate NPSR-like and CCAPR. A site-specific analysis between the vertebrate NPSR and the well studied paralogous vasopressin-like receptor subtypes revealed several putative amino acid sites that may account for the observed functional divergence between them. The data can facilitate experimental studies aiming at deciphering the common features as well as those related to ligand binding and signal transduction processes specific to the NPSR.

## Introduction

The neuropeptide S receptor (NPSR, formerly GPR154), a seven transmembrane spanning G-protein coupled receptor (GPCR) is activated by an endogenous 20 amino acid peptide known as neuropeptide S (NPS) [1]–[4]. The NPSR is widely distributed throughout the central nervous system (CNS) [3], [5]. NPSR mRNA expression is present in many regions in the brain that are associated with regulation of the stress response, memory, the olfactory system and regulation of arousal. In contrast, NPS precursor mRNA is found in isolated cells of the amygdala and the dorsomedial hypothalamic nucleus and especially confined to specific regions of the brainstem including the Barrington's nucleus in the principal sensory trigeminal nucleus, the lateral parabrachial nucleus and a previously undescribed area adjacent to the locus coeruleus (peri-LC) [6]. NPS binds to NPSR with high affinity and activates both G_q_ and G_s_ proteins, leading to increase in free intracellular calcium and cyclic adenosine monophosphate (cAMP) accumulation in cell lines that express NPSR [3], [7], [8]. Very little is known regarding the biochemical and physiological roles of the NPS-NPSR system. Functionally, central dispensation of NPS produces strong anxiolytic-like behavior, increase in wakefulness and locomotor activity and enhances spatial memory and produces anti-nociceptive effects to thermal stimuli in mice [3], [9]–[14]. In line with NPSR expression in the hypothalamus, a key brain region for the regulation of food intake, a recent report demonstrated an inhibitory effect of NPS given intracerebroventricularly on food intake in rats [10].

Multiple isoforms of human NPSR have been reported as products of alternative splicing of NPSR mRNA [2]. Seven transmembrane topology characteristic of GPCRs is encoded only by three isoforms of NPSR variants, of which two variants produce functional receptors that are trafficked to the cell membrane, as demonstrated by current evidence [15]. Multiple single nucleotide polymorphisms (SNPs) have also been identified in human NPSR receptor gene and those are associated with risks of asthma and bronchial hyper-responsiveness [2], [16], [17]. Moreover, the NPSR gene was recognized as a representative gene for specific haplotypes in the human NPSR locus that have been associated with a number of allergic or immunological disorders such as rhinoconjunctivitis, respiratory distress syndrome and irritable bowel syndrome [18]–[20]. For instance, one SNP leading to an Asn/Ile exchange in NPSR results in a 5 to 10-fold increased agonist sensitivity without affecting binding affinity [17], [21], [22]. Potent NPSR antagonists identified recently blunt NPS-mediated arousal and anxiolytic-like effects and might have clinical applications in the treatment of obesity, hypersomia and anxiety disorders without causing sedation [14], [22]–[24].

A previous bioinformatic analysis of NPS sequences revealed that the NPS precursor is highly conserved and is present in all vertebrates with the exception of the ray-finned fish (Actinopterygii). The NPS thus appeared to be specific to tetrapods, including mammals, birds, reptiles and amphibian [25]. However, a novel family of neurophysin-associated neuropeptides (NG peptides), was recently discovered in invertebrate deuterostomes but not in vertebrates, urochordates, protostomes or cnidarians. Interestingly, the NG peptides, so called because of a conserved sequence motif NG, share strong sequence similarity to the N-terminal region of NPS and is suggestive of a probable evolutionary link between the NG peptides and the NPS [26]. The NPSR, also formerly named vasopressin receptor-related receptor (VRR1), is a recently deorphanized GPCR with limited knowledge of the mode of evolution and its divergence from other neuropeptide receptors. NPSR orthologs have been identified in several tetrapod genomes and is consistent with the evolution of its ligand NPS. The closest vertebrate homologs of NPSR are the vasopressin-like receptors. For instance, in a BLAST search, the human NPSR shares about 28 to 34% amino acid sequence identity with the human vasopressin-like receptor subtypes. In this study, using comparative sequence analyses, we sought to: 1) identify NPSR homologs across the metazoan genomes, 2) address the phylogenetic relationship of NPSR with other known neuropeptide receptors to follow the origin and evolution of the NPS associated receptor system and 3) analyze the sites responsible for the evolution of functional divergence between the NPSR and the relatively well studied paralogous vasopressin-like receptor sequences.

## Methods

### Identification of NPS receptors

All known neuropeptide S receptor sequences from the GPCR Database, GPCRDB version 11.3.4 (http://www.gpcr.org/7tm/) [27], UniProt–Protein Knowledgebase (UniProtKB (http://www.uniprot.org/) and Ensembl (http://www.ensemblgenomes.org/) were first obtained. These sequences were used to build a profile using hidden Markov model (HMM) program HMMBUILD and was calibrated using HMMCALIBRATE. This HMM profile was used as a query in the HMMSEARCH program (E value cut off = 1e-5) to search the 65 proteomes downloaded from Ensembl and Joint Genome Institute (JGI) (http://www.jgi.doe.gov/) databases. All HMM based programs were run locally using the HMMER suite of programs with default parameters [28]. Simultaneously, a BLAST search (E value cut-off = 1e-5) was performed using confirmed sequences against UniProtKB and the non-redundant NCBI database. Additionally, the HMMSEARCH against non-redundant NCBI and UniProtKB database was performed using Mobyle Portal server (http://mobyle.pasteur.fr/cgi-bin/portal.py) [29]. Assignment of NPSR orthology for putative hits obtained from the HMMSEARCH and BLAST searches was considered if it had known NPSRs as top 5 hits in a reciprocal BLAST search of the entire GPCRDB. The retrieved NPSR sequences were refined using Cluster Database at High Identity with Tolerance (CD-hit) program, with a word size of 5 and 95% identity as the clustering threshold to remove redundant sequences and pseudogenes [30]. The sequences were examined for transmembrane helices using the TMHMM program (http://www.cbs.dtu.dk/services/TMHMM/) [31]. Sequences having 7 transmembrane helices and those with 6 transmembrane helices were retained assuming that either the last transmembrane might be shorter or missing, while the remaining sequences were removed. The sequences were further manually scrutinized to eliminate isoforms resulting from gene splice variants. Out of a total of 42 sequences, several sequences contained stretches of undetermined residues as a result of incomplete genome information or possible errors in the automated splice site prediction methods used (Table S1). The sequences from lizard and dolphin were incomplete at the N- terminal, while the acorn worm sequence was missing the C- terminal region. The lancelet sequence was truncated at both the N- and C- termini. In this study, these sequences were manually corrected to include missing regions. The missing regions in each case was identified by the use of translated alignments against their genomic regions where the missing region was expected to be found based on alignment with the human N- and C- termini. The final dataset contained 26 sequences of NPSR homologs from 26 organisms. Protein sequences used in this study including the manually corrected sequences are provided in Data S1.

### Multiple sequence alignment and phylogenetic tree construction

Multiple sequence alignment was generated for the final dataset of NPSR sequences and with representatives from other neuropeptide family receptors using MAFFT program, (http://align.bmr.kyushu-u.ac.jp/mafft/online/server), with BLOSUM62 as the scoring matrix and using option G-INS-I for better accuracy for the data set with global homology [32]. The pairwise sequence identities were calculated from the multiple sequence alignments over the entire alignment using the Geneious program [33]. Alignments of the sequences along with secondary structures were displayed using ESPript 2.2 (http://espript.ibcp.fr) [34]. Phylogenetic trees were constructed using Maximum likelihood (ML) and Bayesian methods. Maximum likelihood approach used to infer phylogeny was implemented in MEGA version 5.0 [35]. Evolutionary model and parameters appropriate for phylogeny was determined using ProtTest based on the Akaike Information Criterion (minAIC) [36]. Jones-Taylor-Thornton amino acid substitution matrix with frequency model along with gamma distributed with invariant sites for rate among sites (JTT+I+G+F) was obtained as the best model to determine the evolution for this data set. Results that emerged in ProtTest were consistent with the MEGA substitution model estimation. Robustness of tree topology was measured by testing the phylogeny with 500 bootstrap replications and default parameters were employed for rates, data subset and tree interference options.

Phylogenetic analysis using the Bayesian approach was performed by using MrBayes 3.1.2 [37] with gamma-distributed rate variation and a proportion of invariant sites with frequencies, using JTT model. Markov Chain Monte Carlo (MCMC) analysis was used to approximate the posterior probabilities of the trees. Analysis was run for 3000000 generations and every hundredth tree was sampled. A stop rule was applied to terminate the MCMC generations and convergence of MCMC was assessed until the average standard deviation of split frequencies was dropped below 0.01 (stop value). The first 25% of sampled trees were disregarded as the *burnin* period so that parameter estimates were only made from data drawn from distributions derived after the MCMCs had converged. A consensus tree was built from the remaining 75% of the sampled trees with *sumt* command using the 50% majority rule method. The *sump* command was used to control so that an adequate sample of the posterior probability distribution was reached during the MCMC procedure. The phylogenetic trees were drawn in MEGA v5.0.

### Analysis of gene structure and gene order

Gene structure analysis was carried out using Gene Structure Display Server (GSDS) [38] (http://gsds.cbi.pku.edu.cn/). Coding sequences and the corresponding genomic sequences were submitted to the GSDS for generating the exon-intron map with the intron phase information. The results from the server were checked for compatibility with the information present for the protein sequences in the Ensembl and JGI databases. The analysis included a total of 24 NPSRs, 2 NPSR-like sequences and representatives of the invertebrate CCAPRs and the vasopressin-like receptor family for which gene structure information were available. Intron positions and phases were mapped onto the protein sequence alignment. Intron positions were deemed conserved across the alignment for small changes in intron positions (±10 amino acid residues) [39]. For several truncated sequences that were manually corrected, including CCAPRs, identification of intron positions were carried out using the online splice site prediction server, SplicePredictor [40]. Analysis of synteny was performed by manual examination and comparison of chromosomal loci using genome browsers in the NCBI and Ensembl databases.

### Estimation of functional divergence

Functional divergence analysis between the vertebrate NPSR and its closest vertebrate paralogs, the vasopressin-like receptor family, was performed using Diverge2 program [41]. This method is based on maximum likelihood procedures to estimate significant changes in the site-specific shift of evolutionary rate after the emergence of two paralogous sequences. Sites displaying Type I and Type II functional divergence were identified using the program. Type I divergence between two paralogous groups result in amino acid sites that are highly conserved in one and are variable in the other. In Type II divergence, amino acid sites are highly conserved within the groups but have radically different properties between the groups. Functional divergence tests are based upon the coefficients of divergence (θ), which is the probability that a specific site has diverged in a pairwise comparison. A θ value significantly greater than zero, indicates functional divergence. A posterior probability analysis was used to identify individual sites likely contributing to functional divergence [42]. The cut-off value for the posterior probability was determined by consecutively eliminating the highest scoring sites from the alignment until the θ value dropped to close to zero.

## Results and Discussion

### Identification and distribution of the neuropeptide S receptor

A total of 40 NPSR sequences were identified in vertebrates, including 35 from mammals, 3 from birds and 1 each from reptilian and amphibian species. NPSR orthologs were not detected in several fish genomes including the ray-finned fishes, the elephant shark (*Callorhinchus milii*), a cartilaginous fish that represents the earliest jawed vertebrates, and the sea lamprey (*Petromyzon marinus*), a jawless fish that represents the earliest extant primitive vertebrates. Furthermore, the NPSR could not be detected in invertebrates including the mollusks, ascidians, annelids, insects or cnidaria. These observations are consistent with an earlier study that reported the existence of the NPS peptide only in the tetrapods [25]. Interestingly, sequence searches indicated strong homology of the NPSR to the invertebrate cardioacceleratory peptide receptors (CCAPR). In fact, in all BLAST and HMM searches using a NPSR query sequence, the CCAPRs were among the top non-NPSR hits along with the vertebrate vasopressin-like receptors. The human NPSR shares a sequence identity of about 44% with the *Drosophila melanogaster* CCAPR. The vasopressin-like receptor subtypes include the vertebrate vasopressin 1A (V1AR), vasopressin 1B (V1BR), vasopressin 2 (V2R) and the oxytocin (OTR) receptors and their invertebrate orthologs. In multiple sequence alignments carried out using representative homologs, the NPSRs share pairwise sequence identities in the range 21 to 28% and 22 to 36%, with the vasopressin-like receptor subtypes and the CCAPRs, respectively, over the entire sequence. However, NPSR shares better homology when only the transmembrane regions were compared, with identities in the range 23 to 31% and 30 to 43%, with the vasopressin-like receptor subtypes and the CCAPRs, respectively. It is noteworthy that two NPSR-like sequences were identified in a cephalochordate (*Branchiostoma floridae*) and a hemichordate (*Saccoglossus kowalevskii*). The two invertebrate deuterostome NPSR-like sequences were considered for further analyses since the reciprocal BLAST hits approach used in this study indicated unambiguous one-to-one orthology to vertebrate NPSRs. The sequence identities of lancelet (*B. floridae*) and acorn worm (*S. kowalevskii*) NPSR-like sequences against the human NPSR are about 49% and 38%, respectively. If the N- and C-terminal regions were excluded, the identities of the lancelet and acorn worm sequences increased to 56% and 46%, respectively. The annotation of all putative homologs was subsequently confirmed using phylogenetic analysis as described in the next section.

### Phylogenetic analysis

**Figure 1:**
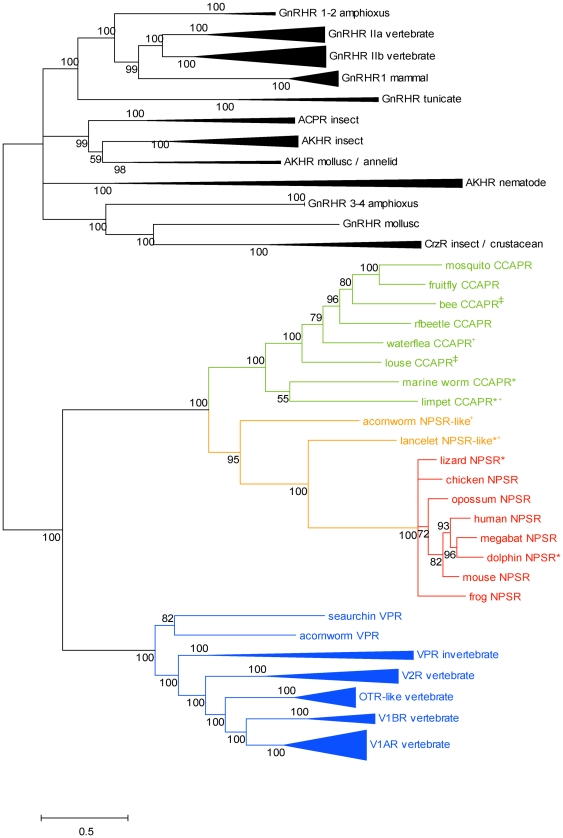
Phylogenetic relationship of the NPSR, CCAPR, GnRHR and vasopressin-like receptors from vertebrates and invertebrates. Bayesian tree of NPSR (red), invertebrate NPSR-like receptor (orange), CCAPR (green), V1AR (blue), V1BR (blue), OTR (blue), V2R (blue), GnRHR (vertebrate and invertebrate Gonadotropin releasing hormone receptor) and VPR (invertebrate vasopressin-like receptor) (blue) sequences. The tree was generated using the Bayesian approach in MrBayes 3.1.2 using JTT+F+I+G model. Bayesian posterior probabilities are marked near branches as a percentage and are used as confidence values of tree branches. Nodes were compressed to represent the animal lineages. Scale bar represents the number of estimated changes per site for a unit of branch length. The receptor group abbreviations, names and accession numbers of the sequences and common and binomial names of the species are as listed in Table S2. In this figure and in Figure 4, the sequence names marked with * and +symbols represent manually corrected sequences at the N terminus and C terminus, respectively. Sequence names marked with ^‡^ symbol in this figure represent fragmented sequences.

Phylogenetic reconstruction using the Bayesian method was employed to investigate the phylogenetic relationships of the NPSR with other peptide receptors of the Rhodopsin family. We used a diverse selection of receptors including the vertebrate and invertebrate vasopressin-like receptor orthologs, the invertebrate CCAPR, vertebrate and invertebrate Gonadotropin-releasing hormone receptor (GnRHR) orthologs and several related neuropeptide receptors. The phylogeny indicated that the tree had two major clades with the vasopressin-like receptor, CCAPR and NPSR clustered into one group (100% confidence value) and all other peptide receptors forming the second cluster, serving as an outgroup (Figure S1, Table S2). This phylogenetic grouping is consistent with a recent evolutionary analysis of the GnRHRs demonstrating that the group of vasopressin-like receptors and the CCAPR form a monophyletic family which is phylogenetically the closest to the GnRHR superfamily, while the related peptide receptors are located basal to this group. However, the study did not include the NPSR [43]. The Bayesian tree that was used for all further analyses in our study contained the NPSR, the vertebrate and invertebrate vasopressin-like receptors, the CCAPR and the GnRHR orthologs. The tree indicated a topology wherein each receptor type converged to form well defined clusters with high confidence values. The GnRHR cluster was used as an outgroup (Figure 1). The major clades include the known vertebrate NPSR and the invertebrate CCAPRs. The vasopressin-like receptors including the vertebrate and invertebrate orthologs form a distinct cluster (100% confidence value) and are located basal to the NPSR/CCAPR group. The subclades within the vasopressin-like receptor clade were in clear agreement with the known receptor specific functional differences and phylogeny described for the four vertebrate subtypes and the invertebrate orthologs (Conopressin, Inotocin, Isotocin, Annetocin, Mesotocin and Cephalotocin receptors) of this large group [44], [45]. An identical topology was observed for the phylogenetic tree obtained using the maximum likelihood approach (Figure S2).

The tree also supports the orthology of the putative NPSR-like receptors identified in the lancelet and acorn worm. These sequences are monophyletic with the vertebrate NPSR cluster with good confidence value of 100% and 95%, respectively. So far the NPSR has been reported to be specific to the tetrapods. However, identification of NPSR-like sequences in lancelet and acorn worm indicates that the common ancestor for a NPSR-like gene might date back to the emergence of the deuterostomes. It is significant that the lancelet and the acorn worm are the closest extant relatives of the chordates and that the topology of the NPSR/NPSR-like cluster follows the expected topology of the ‘tree of life’. Our annotation of the two invertebrate sequences as NPSR-like is further supported by the recent discovery of the NG peptide family [26]. The NG peptide (NGFFFamide-like) and the vasopressin/oxytocin-like peptide precursors share a common domain organization that comprises of the neuropeptide domain followed by a C-terminal neurophysin domain although the NG peptide is structurally unrelated to the cyclic, amidated vasopressin/oxytocin-like peptide. However, the NPS precursor does not contain the C-terminal neurophysin domain and the NPS is not cyclic or amidated. Furthermore, the origin of the vasopressin/oxytocin-like peptide precursors and their receptor systems can be dated back to a common ancestor of bilateral animals, 640–760 million years ago [45]–[48]. In contrast, the NG peptide precursors exist in the lancelet and the acorn worm, but not in cnidarians, protostomes, urochordates or vertebrates, whereas the NPS precursors are specific to the tetrapods [25], [26]. Strikingly, the N-terminal of the human NPS peptide (SFRNGVGTGMKKTSFQRAKS), which is critical for its biological activity, shares sequence identity with the NG peptides (SFRNGVamide in the lancelet and NGFYNamide in the acorn worm) suggesting that the NG peptides may be invertebrate homologs of the vertebrate NPS [26]. One hypothesis that follows is that the lancelet and acorn worm NPSR-like sequences identified in our study are putative receptors for the invertebrate NG peptides. It can be speculated that the relationship within the NPSR/NPSR-like group might be a case of receptor-ligand coevolution wherein the invertebrate NPSR-like sequences have evolved to recognize the NG peptide, while the evolutionarily related vertebrate NPSR recognizes the NPS peptide. Absence of the gene encoding for NPSR-like sequences in urochordates and in marine vertebrates including agnathans, chondrichthyes and teleosts suggests the loss of gene in multiple lineage. The other hypothesis is that the NPS/NPSR system in the vertebrates and NG peptide/NPSR-like system in the invertebrates are indeed homologous, although the physiological functions and peptide domain organization have diverged and the system as a whole or partly, was lost in some lineages. This hypothesis is also possible since no species seem to have both the NPS and NG peptide systems. An alternative explanation is that a specialized NPSR first evolved in the tetrapods and is related to the invertebrate NPSR-like sequences by convergent evolution. Based on the available data, it is not possible to derive conclusions on the nature of relationships between the invertebrate NPSR-like and vertebrate NPSR or on the origin and the common ancestor of the NPSR. While the availability of more basal chordate genomes may aid in obtaining further insights on this issue, a clear experimental demonstration of the functional cross reactivity of the receptors will provide further support for the assumption of ligand-receptor coevolution in the NPSR-NPS/NPSR-like-NG peptide system. There are several clear examples of peptide receptor/ligand coevolution in the vertebrates and in the insects, where an ancestral receptor and ligand gene duplicate several times followed by mutations and evolutionary selection leading to different signaling systems [44], [49]–[52].

### Conserved residues in the NPSR/NPSR-like sequences

**Figure 2:**
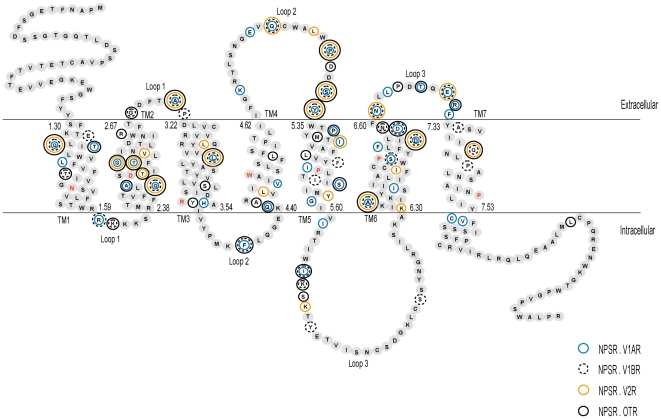
Schematic diagram of the human neuropeptide S receptor. The sequence is drawn and labeled according to the extracellular, intracellular and transmembrane regions. The boundaries of the three regions were based on the definition of these regions for human NPSR [GenBank: NP_997055] given by the Ballesteros-Weinstein nomenclature and TMHMM program. The most conserved residue in each transmembrane helix is denoted with red text. The first and last amino acid residue numbers in each helix is indicated using Ballesteros-Weinstein numbering scheme. Residues that represent sites of functional divergence between the NPSR and the V1AR, V1BR, V2R and OTR subtypes are marked with outlined circles. Residue-wise functional divergence of NPSR with each subtype of vasopressin-like receptor is provided in Data S3.

**Figure 3:**
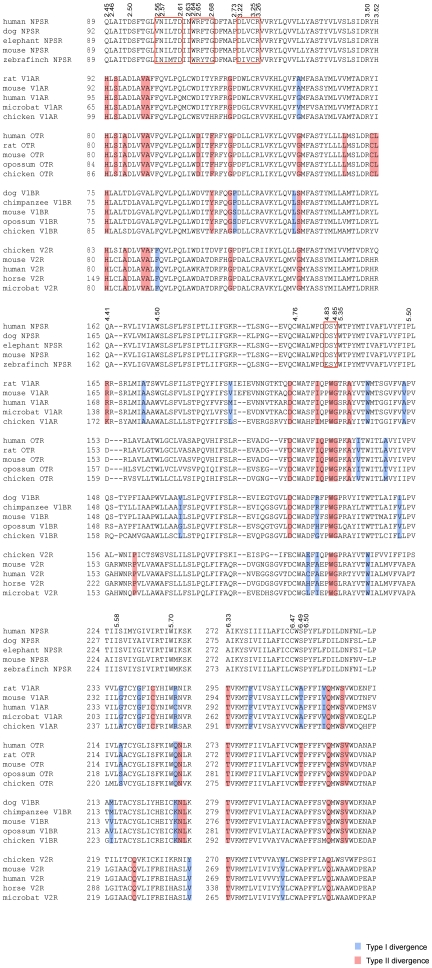
Multiple alignment of functionally divergent sites in the NPSR and vasopressin-like receptors. Samplings of selected functional divergence-related positions in the region starting from TM2 to the end of ECL3 are shown. Amino acid positions (marked on top) are identified by Ballesteros-Weinstein numbering corresponding to the residue position in human NPSR. Contiguous blocks of conserved residues in the NPSR are shown within hollow boxes. Residues associated with Type I and II divergence are marked in blue background and red background, respectively (Data S3).

The NPSR/NPSR-like sequences range from 370 to 394 amino acids in length. The two NPSR-like sequences share pairwise sequence identities in the range 35–40% across the entire sequence and 40 to 52% in the transmembrane helices (TM), in a multiple alignment with NPSR homologs. Several conserved sequence motifs that may play an important role in function were identified based on alignment of the identified sequences. Since the structure-function relationship in the receptor is very poorly studied, a comparison of regions in the sequence corresponding to known functional regions in the better-studied paralogous vasopressin-like receptor sequences was carried out to identify common as well as selective receptor positions. In all subsequent analyses and comparisons of residue positions, the residue numbering includes Ballesteros-Weinstein numbers in superscript [53], [54]. The topology of the regions comprising the three structural parts, namely the TM, the extracellular loops (ECL) and the intracellular loops (ICL) have been assigned from the prediction of TM regions for the human NPSR entry [GenBank: NP_997055] using the TMHMM program (Figure 2, Figure S3). Examples of common peptide GPCR conserved motifs include LxxxD (90^2.46^–94^2.50^) in TM2, CWxP (286^6.47^–289^6.50^) in TM6, NPxxY (326^7.49^–330^7.53^) in TM7. Cysteine residues (C121^3.25^, C197^4.76^) responsible for the formation of disulfide bond between extracellular loops (ECL1 and 2) are also conserved [55]–[57]. Examination of the sequence alignment indicates that these positions are completely conserved in the NPSR/NPSR-likes and the vasopressin-like receptors. Two conserved motifs WXFG and DXXCR in ECL1 have been reported to be crucial for ligand binding and signaling in the neuropeptide receptors [58]. In the vertebrate NPSR, these regions correspond to highly conserved WRFTG (108^2.64^–112^2.68^) and DLVCR (118^3.22^–122^3.26^) motifs (Figure 3). However, the NPSR-like sequences appear to have a modified HRFTX motif in place of WRFTG. Other functionally important regions for agonist recognition in the vasopressin-like receptors includes the completely conserved regions FQVLPQ at the end of TM2 and motif PWG in the ECL2 [56], [59], [60]. However, the corresponding regions in the NPSR contains different, albeit highly conserved motifs VNILTD (100^2.56^–105^2.61^) and DSY (204^4.83^–206^4.85^), respectively (Figure 3).

Known functionally important sites specific to NPSRs include N101^2.57^, wherein a N101A mutation led to incomplete glycosylated forms of the receptor protein without affecting function and cell surface expression. However, biogenesis of the receptor could be affected by the altered conformation caused due to the change at N101^2.57^ [58]. This site is completely conserved. Additionally, structure-activity studies have shown that Asp at position 105^2.61^ could play a direct role in agonist binding by ionic interactions with a conserved Arg residue at position 3 in the NPS peptide [22], [58]. A negatively charged residue, D/E at this position, is also completely conserved across the alignment. Site-directed mutagenesis and transient expression studies on NPSR indicated that mutation of residue N107^2.63^ to Ile, associated with asthma susceptibility, led to an increase in potency and maximal efficacy of NPS due to higher level of cell surface receptor density of mutant compared with wild type receptor [21]. Examination of the sequence alignment indicates that the human sequence is a natural variant with an Asn at this position whereas it is Ile in all other sequences (Figure 3, Figure S3). Thus, sequence conservation patterns in the NPSR are consistent with previous knowledge of functional sites and suggest additional examples of group specific residues.

### Gene structure and order analysis

**Figure 4:**
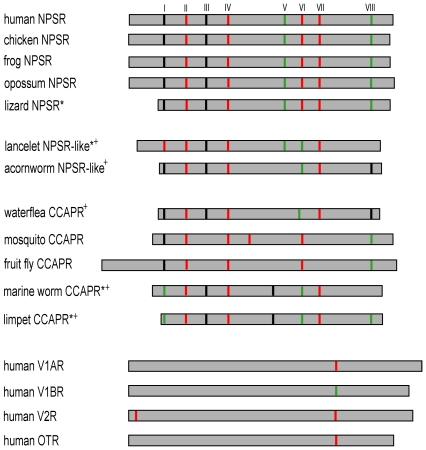
Conservation and variability of intron positions and phases. Schematic of the multiple alignment of amino acid sequences of the NPSR, NPSR-like and representatives from the CCAPR and vasopressin-like receptor subtypes are shown. The 0, 1, 2 phase introns are marked with black, red and green lines, respectively. Introns corresponding to human NPSR are named I to VIII according to their positions in the amino acid sequence. The gene structure details of all sequences indicating exon-intron lengths, intron positions and phases are presented in Figure S3, S4 and Data S2.

Gene structure features may aid in supporting the phylogenetic inference since conservation of exon-intron structure has been reported in clades of orthologous genes and in families of paralogous genes and protein superfamilies [61]. A comparative analysis of the genomic structures of the NPSR, NPSR-like, CCAPR and the vasopressin-like receptor was carried out (Figure S3, Figure S4, Data S2). Figure 4 illustrates the distribution, position and phase of introns within the amino acid sequences. In general, the NPSR genes are composed of 9 coding exons. In order to assist comparisons, the introns were named as I to VIII according to the inserted positions in human NPSR. The positions of the 8 introns in NPSRs are highly conserved as expected. The invertebrate NPSR-like sequences are composed of 7 exons. Thus it appears that the NPSR-like and NPSR sequences contain similar number of introns. Intron V and intron VIII are lost in the acorn worm and the lancelet, respectively. Another variation between the NPSR-like and NPSR include phase change in intron VI to phase 2 in the NPSR-like. It is noteworthy that both intron positions and phases in the NPSR-like are highly conserved to that in the NPSRs. The CCAPRs contain a mostly conserved set of 6 to 9 exons [62]. Remarkably, several intron positions and phases are highly conserved across the NPSR, NPSR-like and the CCACPR (introns I, II, III, IV, VI, VII and VIII). The few intron loss or gain events that occur in these sequences appear to be dependent on lineage-specific events. The vasopressin-like receptors are mostly composed of 2 to 3 exons [56], [63], [64]. The position of the single intron in human V1AR is the most conserved among this group of receptors. This position is about 25 residues away from intron VII of the human NPSR. Thus, conserved intron gain relative to the vasopressin-like receptors appears to be a conserved feature of the NPSR/NPSR-like/CCAPR group of receptors. The presence of relatively larger number of introns could have a role in alternative splicing events, transcriptional regulatory events and exon shuffling [65]–[67]. Several alternatively spliced GPCR variants with different TM topology have been shown to exist, although their biological functions are elusive [68], [69]. However, studies with GnRHR isoforms suggest that the truncated variants may inhibit the signaling or transport of the wild-type receptor [70]. At least two alternatively spliced forms of human NPSR have been identified and found to show different pharmacological profiles [15].

Remarkably, the gene structure-inferred view of the NPSR/NPSR-like/CCAPR group of the receptors thus shares basic topological features with the protein sequence based phylogeny. It is clear that the NPSR is related to the NPSR-like and the two receptors are most likely orthologous, although the sampling of NPSR-like may be insufficient. Furthermore, it is intriguing that the CCAPR, predominantly identified in the arthropods so far, should show congruence in their relationship to the NPSR/NPSR-like group at the level of the protein sequence and the gene structures. Assuming that the pattern of intron-exon boundaries are good markers of the history of descent of conserved gene families, one interpretation from our analyses, albeit highly speculative, is that the CCAPR is an ortholog of the NPSR/NPSR-like genes. Further, it follows that the common ancestor to this receptor group might date back to an ancestral bilateral animal, concomitant with the emergence of the vasopressin-like receptors. Finally, an analysis of the gene order of the chromosomal region containing the NPSR gene revealed the expected conserved synteny of the NPSR gene loci across the vertebrates. However, the NPSR-like and CCAPR genes do not display conserved synteny to the NPSR.

### Divergence between vertebrate NPSR and vasopressin-like receptors

Next, we used an analysis of position-specific rate shift variation to identify putative amino acid residues that may have contributed to specific sequence–structural features that distinguish the vertebrate NPSRs from their closest vertebrate neuropeptide homologs, the vasopressin-like receptors. Although the ligands are unrelated, the vasopressin-like receptors were chosen as the closest paralog of the NPSR for the following reasons. Firstly, the large scale phylogenetic analysis carried out here indicate that the vasopressin-like receptors are indeed the phylogenetically closest group to the NPSR, suggesting a common ancestry with deep roots. Additional evidence comes from chemogenomic analyses of human non-olfactory GPCRs which indicate that the NPSR and the vasopressin-like receptors are clustered in a strongly supported group, independent of the other peptide receptors [71], [72]. The dendrograms in these analyses used alignments of critical residues (∼30–45 residues) in the TM-binding cavity following the principle that similar ligands are accommodated by similar binding pockets. In both studies, the clustering topology for the receptors reflects ligand affinities thus suggesting strong physicochemical homology of the TM-binding cavity between the NPSR and the vasopressin-like receptors [71], [72]. Finally, Gupte et al., 2004, showed typical GPCR signaling by a chimeric receptor, V1AR/NPSR, which contained the human V1A receptor with all three intracellular loops (ICLs) and C terminus replaced by those of NPSR. The chimeric receptor was functionally responsive to vasopressin. Furthermore, the engineered construct behaved like a typical GPCR when expressed in mammalian cell lines and point mutations made in TM2 were shown to drastically affect activation. The chimeric receptor created to test signaling of NPSR was based on the homology of the two receptors and showed properties including dual signaling by coupling to both G_q_ and G_s_ pathways, consistent with the activation of NPSR by NPS [8]. The signaling properties of the chimera cannot be ascribed solely to the presence of the TMs and the ECLs of the V1A receptor.

An amino acid residue in a cluster of homologous sequences is deemed functionally important if it is evolutionarily conserved. Therefore, changes in selective constraints on particular amino acid sites can be used as a measure of functional divergence of duplicated genes [42]. Type I functional divergence refers to the evolutionary process where after duplication, the evolutionary rate at an amino acid site may increase for functional divergence-related change in the early stage resulting in related but distinct functions between the two gene clusters. Typically, in Type I divergence, an amino acid residue is highly conserved in one cluster, but highly diverse in the other one, implying that these sites have undergone altered functional constraints (i.e., different evolutionary rates). In contrast, Type II functional divergence between the gene clusters does not result in altered functional constraints but a radical change in amino acid property between them (for instance, hydrophobicity, charge, etc). In a typical Type II case, the homologous site in a duplicate gene cluster is evolutionarily conserved within each of the clusters while drastic shift in amino acid property has occurred between the clusters. Such cluster-specific sites have most likely undergone purifying selection in the late phase after gene duplication resulting in cluster specific functional properties. The estimated coefficient of functional divergence between duplicate genes is defined as the probability of being related to functional divergence, with a large value indicating a high level of divergence. Furthermore, a site-specific profile based on empirical posterior analysis can be used to predict residues that are likely to be responsible for functional divergence between the two clusters [42], [73], [74].

In this study, a set of 24 NPSR sequences and 20 sequences each from the four vertebrate vasopressin-like receptor subtypes (V1AR, V1BR, V2R and OTR) were used to estimate the coefficient of Type I and II functional divergence in four separate comparisons. Estimates of coefficients of divergence (θ) indicate that the NPSR and vasopressin-like receptor group have indeed undergone diversification (Table 1, Data S3). The θ values for Type I and II divergence between the NPSR and vasopressin-like receptor subtypes are significantly larger than zero (Table 1). In contrast, the θ values between the vasopressin-like receptor subtypes range from 0.32–0.47 (p<0.0001) for Type I and are not significant for Type II, indicating relatively lower levels of function divergence between the vasopressin-like receptor subtypes (data not shown). The predicted sites that contribute to divergence were mapped onto three structurally distinct regions; TM regions, the ECLs and the ICLs (Figure 2). A total of 51 Type I divergence sites with posterior probability ratios ranging between 0.90–0.99 were identified (Data S3). The data indicate that Type I sites are distributed across all three regions and have distinct patterns of distribution for each NPSR-vasopressin-like receptor subtype comparison (Table 1). Furthermore, examination of conservation patterns in the alignment shows that the Type I sites involved sets of divergent and conserved sites in both groups suggesting that the early phase of duplicate evolution continued after the first duplication into paralogous groups. These findings suggest that site-specific changes may have occurred because of relaxation of selective constraints in the sequences of either group. Alternatively, the conserved sites in either group are indicative of newly acquired functional constraints that were not present in an ancestral neuropeptide receptor gene. Among the predicted sites, two have known pharmacological relevance. For example, Ser288^6.49^ in human NPSR is a significant residue in the four residue motif CWSP located in TM6 [64]. Missense mutation of the corresponding residue (A285P) in human V2R was identified to cause X-linked nephrogenic diabetes insipidus [64], [75]. Alanine at this position is highly conserved in V1AR, V1BR and V2R (Figure 3). This Type I site was recognized with a posterior probability of 0.957 and was conserved in the NPSR (Data S3). Type II divergence sites most probably encode paralog-specific properties since these modifications occur by adaptive fixation of variants in either groups. The total number of Type II sites (88 residues) increased significantly compared to Type I sites. These sites are mostly distributed across the ECLs and the TM regions. This increase in sites suggests a more pronounced role of adaptive evolution in the late phase after duplication and divergence of these receptors. Some of the predicted Type II sites correspond to residues with experimentally characterized functional role for agonist or antagonist binding or for receptor activation in the vasopressin-like receptors (Figure 3). For instance, the conserved G112^2.68^ located in the ECL2 in human NPSR corresponds to Y115 in V1AR, D103 in V2R and F102 in OTR in pairwise comparisons. This site in the vasopressin-like receptors is significant for the selectivity of specific agonist against each subgroup receptor [56], [76]–[79]. Similarly, the residue R109^2.65^ at the extracellular end of TM2 in NPSR is equivalent to residue K100 in the V2R that has been shown to influence the binding of peptide agonist [78]. Besides this, F309^6.70^ located on ECL3 of NPSR is equivalent to G304 in the V2R. G304 has been reported to be involved in stabilizing the conformation of V2R to enable species-selective binding of cyclic peptide antagonists [78] (Figure S3). Similarly, Q225 in V2 receptor, which is critical for G_s_ recognition and activation, corresponds to Y230^5.58^ in NPSR [80]. It is reasonable to hypothesize that several of these predicted functionally divergent sites could possibly play a role in the specific properties of the NPSR.

**Table 1:** Type I and Type II functional divergence between the NPSR and vasopressin-like receptor subtypes.

Divergence	Comparison	θ±SE	z	TM	ECL	ICL
Type I	NPSR . V1AR	0.74±0.07	10.22	11	5	2
	NPSR . V1BR	0.52±0.07	7.84	3	3	6
	NPSR . V2R	0.51±0.07	7.65	5	4	1
	NPSR . OTR	0.63±0.07	9.20	5	1	3
Type II	NPSR . V1AR	0.53±0.06	9.04	12	8	3
	NPSR . V1BR	0.44±.007	6.42	10	6	2
	NPSR . V2R	0.49±.07	6.48	11	3	-
	NPSR . OTR	0.54±0.05	10.4	21	10	1

*Note:* θ denotes the coefficient of functional divergence. SE is standard error. z score is the value of confidence and is obtained by θ/SE. P value is the probability of the z score, which had a value of <0.0001 in all comparisons. The symbol - indicates the absence of divergent sites. Abbreviations: TM – Transmembrane helices, ECL – Extracellular loops, ICL – Intracellular loops.

It must be mentioned here that there is significant overlap of Type I and Type II sites across the four group-wise comparisons, namely, a Type I divergent site in the NPSR.V1AR comparison may represent a Type II site in the NPSR.V2R comparison. A total of 75 unique sites show either one or both types of functional divergence, across the groups (Figure 2). Out of these, 9 sites display divergence across all four subtype comparisons, while 9 sites and 17 sites display divergence across three and two subtype comparisons, respectively. It is very likely that the divergent sites that are common across multiple comparisons are hotspots for changes in evolutionary rates and for selection or relaxation of constraints in the evolution of functional divergence of the NPSR and the vasopressin-like receptors. The percentage of occurrence of divergent sites in each structural domain ranges from 14 to 38% of the total number of residues comprising the respective domain (Data S3). It is noteworthy that out of a total of 39 residues in the ECLs, at least 18 sites display either type of divergence across all comparisons (Data S3). This includes 23 out of 8 residues in ECL2 and 7 out of 10 residues of ECL3. This data is in good agreement with the large volume of published GPCR data which indicate that most of the endogenous ligands of the Rhodopsin family GPCRs bind within the TM domain close to the ECL2 [71], [81]. Out of a total of 194 residues in the TMs, 44 sites display divergence. TM5 contains the largest percentage of divergent sites (10 out of 26 residues), followed by TM6, TM2 and TM4 (9 out of 31 residues, 7 out of 30 residues and 5 out of 23 residues). These residues in the ECLs and TM 2, 4, 5 and 6 may thus be sensitive to correlated residue-ligand binding and/or residue-signal transmission specificities of the NPSR. These observations are also in general agreement with well known experimental data on the structural rearrangements of the TMs 2, 5, 6 and 7 caused by amino acids acting as ‘micro-switches’ during ligand induced activation [82]–[85]. Assuming that the NPSR and the vasopressin-like receptors are paralogs, it can be speculated that the predicted residues that are conserved within the NPSR evolved by selection and could be important for the stability of the structure or act as critical mediators of the signal transmission though the NPSR. Future biochemical studies can be focused on experimental verification of the role of the predicted divergent sites in determining paralog specific properties.

### Conclusions

A NPSR-like sequence has been identified at the emergence of the hemichordate suggesting that the origin of the NPSR-like gene is much older than previously assumed. Comparative analyses at the levels of amino acid sequences and gene structures supported the possible orthology of the NPSR and the NPSR-like, although studies on the cross reactivity of these receptors with peptide ligands are required to validate this assumption. Available data also suggest that the NPSR/NPSR-like is orthologous to the invertebrate CCAPR and the NPSR/NPSR-like/CCAPR group is phylogenetically the most closely related to the group of vertebrate and invertebrate vasopressin-like receptors. Site-specific analysis suggests divergence between the NPSR and the paralogous vasopressin-like receptor and has demonstrated that a majority of functionally divergent sites are located on the transmembrane helices 2, 4, 5 and 6 and at the extracellular loops. We conclude that the specific effect of ligand on NPSR signal transduction may be primarily determined by specific sites or a combination of the sites predicted in this study. The results may be used for the design of agonist binding studies, site-directed mutagenesis and other experiments focused on investigating novel antagonists of NPSR.

## Supporting Information

Figure S1 **Phylogenetic relationship of the NPSR and peptide receptors.** Bayesian tree of NPSR (red), invertebrate NPSR-like receptor (orange), CCAPR (green), V1AR (blue), V1BR (blue), OTR (blue), V2R (blue), VPR (invertebrate vasopressin-like receptor) (blue), the vertebrate and invertebrate GnRHR (gonadotropin releasing hormone receptor), NPFFR (neuropeptide FF receptor), TACR (tachykinin receptor), the vertebrate NMUR (neuromedin U receptor), NTSR (neurotensin receptor), GALR (galanin receptor), KISSR (kisspeptin receptor), NPBWR (neuropeptide W/neuropeptide B receptor), SSTR (somatostatin receptor), NPYR (neuropeptide Y receptor) and the invertebrate AKHR (adipokinetic hormone receptor), ACPR (adipokinetic hormone receptor/corazonin-related peptide receptors), CrzR (corazonin receptor). The tree was generated using the Bayesian approach in MrBayes 3.1.2 using JTT+F+I+G model. Analysis was run for 3000000 generations and every hundredth tree was sampled, until the average standard deviation of split frequencies dropped below the stop value of 0.02. Bayesian posterior probabilities are marked near branches as a percentage and are used as confidence values of tree branches. Nodes were compressed to represent the animal lineages. Scale bar represents the number of estimated changes per site for a unit of branch length. The receptor group abbreviations, names and accession numbers of the sequences and common and binomial names of the species are as listed in Table S2. The sequence names marked with * and +symbols represent sequences manually corrected at the N terminus and C terminus, respectively.
(TIF)

Figure S2 **Phylogenetic relationship of the NPSR, CCAPR, GnRHR and vasopressin-like receptors from vertebrates and invertebrates.** Maximum likelihood tree of NPSR (red), invertebrate NPSR-like receptor (orange), CCAPR (green), V1AR (blue), V1BR (blue), OTR (blue), V2R (blue), GnRHR (vertebrate and invertebrate Gonadotropin releasing hormone receptor) and VPR (invertebrate vasopressin-like receptor) (blue) sequences. The tree was generated using the maximum likelihood method in MEGA5.0 and bootstrap test was carried out with 500 replicates Best-fit model (JTT+I+G+F) was selected by ProtTest v2.4. Numbers on the nodes represents the bootstrap values and the clades possessing less than 50% bootstrap support were not marked. Nodes were compressed to represent the animal lineages. Scale bar represents the number of estimated changes per site for a unit of branch length. The receptor group abbreviations, names and accession numbers of the sequences and common and binomial names of the species are as listed in Table S2. The sequence names marked with * and +symbols represent sequences fragmented at the N terminus and C terminus, respectively. Sequence names marked with ^‡^ symbol in this figure represent fragmented sequences.
(TIF)

Figure S3 **Multiple sequence alignment of NPSR, NPSR-like and representative CCAPR sequences.**
(PDF)

Figure S4 **Gene structure representation of NPSR, NPSR-like and representative CCAPR, V1AR, V1BR, V2R and OTR sequences.**
(PDF)

Table S1 **List of identified neuropeptide S receptors.**
(DOC)

Table S2 **Accession numbers of all protein sequences used in the phylogenetic analyses.**
(DOC)

Data S1 **Amino acid sequences of NPSR and NPSR-like sequences in FASTA format.**
(DOC)

Data S2 **Length of exon-intron regions in the NPSR, NPSR-like and representative CCAPR, V1AR, V1BR, V2R and OTR sequences.**
(XLS)

Data S3 **Functional divergence between NPSR and vertebrate vasopressin-like sequences.** Sheet 1: List of Accession numbers of the vasopressin-like receptor subtype sequences used in analysis of Type I and Type II functional divergence. Sheet 2: Site specific profile for predicting amino acid residues responsible for Type I functional divergence between NPSR and V1AR, V1BR, V2R, and OTR groups, respectively, measured by posterior probability. Sheet 3: Site specific profile for predicting amino acid residues responsible for Type II functional divergence between NPSR and V1AR, V1BR, V2R, and OTR groups, respectively, measured by posterior probability. Sheet 4: Type I functionally divergent sites predicted between NPSR and V1AR, V1BR, V2R and OTR receptor subtypes, respectively and their location in NPSR domain topology. Sheet 5: Type II functionally divergent sites predicted between NPSR and V1AR, V1BR, V2R and OTR receptor subtypes, respectively and their location in NPSR domain topology. Sheet 6: Percentage of occurrence of Type I and /or Type II divergent sites across structural domains of human NPSR.
(XLS)

## References

[1] Laitinen T, Daly MJ, Rioux JD, Kauppi P, Laprise C (2001). A susceptibility locus for asthma-related traits on chromosome 7 revealed by genome-wide scan in a founder population.. Nat Genet.

[2] Laitinen T, Polvi A, Rydman P, Vendelin J, Pulkkinen V (2004). Characterization of a common susceptibility locus for asthma-related traits.. Science.

[3] Xu YL, Reinscheid RK, Huitron-Resendiz S, Clark SD, Wang Z (2004). Neuropeptide S: a neuropeptide promoting arousal and anxiolytic-like effects.. Neuron.

[4] Gloriam DE, Schioth HB, Fredriksson R (2005). Nine new human rhodopsin family G-protein coupled receptors: Identification, sequence characterization and evolutionary relationship.. Biochim Biophys Acta.

[5] Reinscheid RK, Xu YL, Civelli O (2005). Neuropeptide S: a new player in the modulation of arousal and anxiety.. Mol Interv.

[6] Xu YL, Gall CM, Jackson VR, Civelli O, Reinscheid RK (2007). Distribution of neuropeptide S receptor mRNA and neurochemical characteristics of neuropeptide S-expressing neurons in the rat brain.. J Comp Neurol.

[7] Sato S, Shintani Y, Miyajima N, Yoshimura K (2002). Novel G protein-coupled receptor protein and DNA thereof..

[8] Gupte J, Cutler G, Chen JL, Tian H (2004). Elucidation of signaling properties of vasopressin receptor-related receptor 1 by using the chimeric receptor approach.. Proc Natl Acad Sci U S A.

[9] Peng YL, Han RW, Chang M, Zhang L, Zhang RS (2010). Central Neuropeptide S inhibits food intake in mice through activation of Neuropeptide S receptor.. Peptides.

[10] Beck B, Fernette B, Stricker-Krongrad A (2005). Peptide S is a novel potent inhibitor of voluntary and fast-induced food intake in rats.. Biochem Biophys Res Commun.

[11] Pape HC, Jungling K, Seidenbecher T, Lesting J, Reinscheid RK (2010). Neuropeptide S: a transmitter system in the brain regulating fear and anxiety.. Neuropharmacology.

[12] Duangdao DM, Clark SD, Okamura N, Reinscheid RK (2009). Behavioral phenotyping of neuropeptide S receptor knockout mice.. Behav Brain Res.

[13] Han RW, Yin XQ, Chang M, Peng YL, Li W (2009). Neuropeptide S facilitates spatial memory and mitigates spatial memory impairment induced by N-methyl-D-aspartate receptor antagonist in mice.. Neurosci Lett.

[14] Li W, Chang M, Peng YL, Gao YH, Zhang JN (2009). Neuropeptide S produces antinociceptive effects at the supraspinal level in mice.. Regul Pept.

[15] Vendelin J, Pulkkinen V, Rehn M, Pirskanen A, Raisanen-Sokolowski A (2005). Characterization of GPRA, a novel G protein-coupled receptor related to asthma.. Am J Respir Cell Mol Biol.

[16] Nepomuceno D, Sutton S, Yu J, Zhu J, Liu C (2010). Mutagenesis studies of neuropeptide S identify a suitable peptide tracer for neuropeptide S receptor binding studies and peptides selectively activating the I(107) variant of human neuropeptide S receptor.. Eur J Pharmacol.

[17] Bernier V, Stocco R, Bogusky MJ, Joyce JG, Parachoniak C (2006). Structure-function relationships in the neuropeptide S receptor: molecular consequences of the asthma-associated mutation N107I.. J Biol Chem.

[18] Melen E, Bruce S, Doekes G, Kabesch M, Laitinen T (2005). Haplotypes of G protein-coupled receptor 154 are associated with childhood allergy and asthma.. Am J Respir Crit Care Med.

[19] Pulkkinen V, Haataja R, Hannelius U, Helve O, Pitkanen OM (2006). G protein-coupled receptor for asthma susceptibility associates with respiratory distress syndrome.. Ann Med.

[20] D'Amato M, Bruce S, Bresso F, Zucchelli M, Ezer S (2007). Neuropeptide s receptor 1 gene polymorphism is associated with susceptibility to inflammatory bowel disease.. Gastroenterology.

[21] Reinscheid RK, Xu YL, Okamura N, Zeng J, Chung S (2005). Pharmacological characterization of human and murine neuropeptide s receptor variants.. J Pharmacol Exp Ther.

[22] Okamura N, Habay SA, Zeng J, Chamberlin AR, Reinscheid RK (2008). Synthesis and pharmacological in vitro and in vivo profile of 3-oxo-1,1-diphenyl-tetrahydro-oxazolo[3,4-a]pyrazine-7-carboxylic acid 4-fluoro-benzylamide (SHA 68), a selective antagonist of the neuropeptide S receptor.. J Pharmacol Exp Ther.

[23] Camarda V, Rizzi A, Ruzza C, Zucchini S, Marzola G (2009). In vitro and in vivo pharmacological characterization of the neuropeptide s receptor antagonist [D-Cys(tBu)5]neuropeptide S.. J Pharmacol Exp Ther.

[24] Ruzza C, Rizzi A, Trapella C, Pela' M, Camarda V (2010). Further studies on the pharmacological profile of the neuropeptide S receptor antagonist SHA 68.. Peptides.

[25] Reinscheid RK (2007). Phylogenetic appearance of neuropeptide S precursor proteins in tetrapods.. Peptides.

[26] Elphick MR (2010). NG peptides: a novel family of neurophysin-associated neuropeptides.. Gene.

[27] Vroling B, Sanders M, Baakman C, Borrmann A, Verhoeven S (2011). GPCRDB: information system for G protein-coupled receptors.. Nucleic Acids Res.

[28] Eddy SR (1998). Profile hidden Markov models.. Bioinformatics.

[29] Neron B, Menager H, Maufrais C, Joly N, Maupetit J (2009). Mobyle: a new full web bioinformatics framework.. Bioinformatics.

[30] Li W, Godzik A (2006). Cd-hit: a fast program for clustering and comparing large sets of protein or nucleotide sequences.. Bioinformatics.

[31] Krogh A, Larsson B, von Heijne G, Sonnhammer EL (2001). Predicting transmembrane protein topology with a hidden Markov model: application to complete genomes.. J Mol Biol.

[32] Katoh K, Kuma K, Toh H, Miyata T (2005). MAFFT version 5: improvement in accuracy of multiple sequence alignment.. Nucleic Acids Res.

[33] Drummond AJ, Ashton B, Buxton S, Cheung M, Cooper A (2010). http://www.geneious.com.

[34] Gouet P, Courcelle E, Stuart DI, Metoz F (1999). ESPript: analysis of multiple sequence alignments in PostScript.. Bioinformatics.

[35] Tamura KPD, Peterson N, Stecher G, Nei M, Kumar S (2011). MEGA5: Molecular Evolutionary Genetics Analysis using Maximum Likelihood, Evolutionary Distance, and Maximum Parsimony Methods.. Mol Biol Evol.

[36] Abascal F, Zardoya R, Posada D (2005). ProtTest: selection of best-fit models of protein evolution.. Bioinformatics.

[37] Ronquist F, Huelsenbeck JP (2003). MrBayes 3: Bayesian phylogenetic inference under mixed models.. Bioinformatics.

[38] Guo AY, Zhu QH, Chen X, Luo JC (2007). [GSDS: a gene structure display server].. Yi Chuan.

[39] Betts MJ, Guigo R, Agarwal P, Russell RB (2001). Exon structure conservation despite low sequence similarity: a relic of dramatic events in evolution?. EMBO J.

[40] Brendel V, Xing L, Zhu W (2004). Gene structure prediction from consensus spliced alignment of multiple ESTs matching the same genomic locus.. Bioinformatics.

[41] Gu X, Vander Velden K (2002). DIVERGE: phylogeny-based analysis for functional-structural divergence of a protein family.. Bioinformatics.

[42] Gu X (1999). Statistical methods for testing functional divergence after gene duplication.. Mol Biol Evol.

[43] Roch GJ, Busby ER, Sherwood NM (2011). Evolution of GnRH: diving deeper.. Gen Comp Endocrinol.

[44] Hoyle CH (1999). Neuropeptide families and their receptors: evolutionary perspectives.. Brain Res.

[45] Aikins MJ, Schooley DA, Begum K, Detheux M, Beeman RW (2008). Vasopressin-like peptide and its receptor function in an indirect diuretic signaling pathway in the red flour beetle.. Insect Biochem Mol Biol.

[46] Kawada T, Sekiguchi T, Itoh Y, Ogasawara M, Satake H (2008). Characterization of a novel vasopressin/oxytocin superfamily peptide and its receptor from an ascidian, Ciona intestinalis.. Peptides.

[47] Stafflinger E, Hansen KK, Hauser F, Schneider M, Cazzamali G (2008). Cloning and identification of an oxytocin/vasopressin-like receptor and its ligand from insects.. Proc Natl Acad Sci U S A.

[48] Ukena K, Iwakoshi-Ukena E, Hikosaka A (2008). Unique form and osmoregulatory function of a neurohypophysial hormone in a urochordate.. Endocrinology.

[49] Hansen KK, Stafflinger E, Schneider M, Hauser F, Cazzamali G (2010). Discovery of a novel insect neuropeptide signaling system closely related to the insect adipokinetic hormone and corazonin hormonal systems.. J Biol Chem.

[50] Park JI, Semyonov J, Chang CL, Hsu SY (2005). Conservation of the heterodimeric glycoprotein hormone subunit family proteins and the LGR signaling system from nematodes to humans.. Endocrine.

[51] Dreborg S, Sundstrom G, Larsson TA, Larhammar D (2008). Evolution of vertebrate opioid receptors.. Proc Natl Acad Sci U S A.

[52] Sundström G, Dreborg S, Larhammar D (2010). Concomitant duplications of opioid peptide and receptor genes before the origin of jawed vertebrates.. PLoS One.

[53] Ballesteros JA, Weinstein H (1995). Integrated methods for the construction of three dimensional models and computational probing of structure function relations in G protein-coupled receptors.. Methods Neurosci.

[54] Warne T, Serrano-Vega MJ, Baker JG, Moukhametzianov R, Edwards PC (2008). Structure of a beta1-adrenergic G-protein-coupled receptor.. Nature.

[55] Oliveira L, Paiva ACM, Vriend G (1993). A common motif in G-protein-coupled seven transmembrane helix receptors.. Journal of Computer-Aided Molecular Design.

[56] Gimpl G, Fahrenholz F (2001). The oxytocin receptor system: structure, function, and regulation.. Physiol Rev.

[57] Macrae AD, Premont RT, Jaber M, Peterson AS, Lefkowitz RJ (1996). Cloning, characterization, and chromosomal localization of rec1.3, a member of the G-protein-coupled receptor family highly expressed in brain.. Brain Res Mol Brain Res.

[58] Clark SD, Tran HT, Zeng J, Reinscheid RK (2010). Importance of extracellular loop one of the neuropeptide S receptor for biogenesis and function.. Peptides.

[59] Mouillac B, Chini B, Balestre MN, Elands J, Trumpp-Kallmeyer S (1995). The binding site of neuropeptide vasopressin V1a receptor. Evidence for a major localization within transmembrane regions.. J Biol Chem.

[60] Conner M, Hawtin SR, Simms J, Wootten D, Lawson Z (2007). Systematic analysis of the entire second extracellular loop of the V(1a) vasopressin receptor: key residues, conserved throughout a G-protein-coupled receptor family, identified.. J Biol Chem.

[61] Sanchez D, Ganfornina MD, Gutierrez G, Marin A (2003). Exon-intron structure and evolution of the Lipocalin gene family.. Mol Biol Evol.

[62] Cazzamali G, Hauser F, Kobberup S, Williamson M, Grimmelikhuijzen CJ (2003). Molecular identification of a Drosophila G protein-coupled receptor specific for crustacean cardioactive peptide.. Biochem Biophys Res Commun.

[63] Zingg HH (1996). Vasopressin and oxytocin receptors.. Baillieres Clin Endocrinol Metab.

[64] Oksche A, Rosenthal W (1998). The molecular basis of nephrogenic diabetes insipidus.. J Mol Med (Berl).

[65] Majewski J, Ott J (2002). Distribution and characterization of regulatory elements in the human genome.. Genome Res.

[66] Qiu WG, Schisler N, Stoltzfus A (2004). The evolutionary gain of spliceosomal introns: sequence and phase preferences.. Mol Biol Evol.

[67] Sverdlov AV, Rogozin IB, Babenko VN, Koonin EV (2005). Conservation versus parallel gains in intron evolution.. Nucleic Acids Res.

[68] Schwarz DA, Barry G, Eliasof SD, Petroski RE, Conlon PJ (2000). Characterization of gamma-aminobutyric acid receptor GABAB(1e), a GABAB(1) splice variant encoding a truncated receptor.. J Biol Chem.

[69] Vanetti M, Vogt G, Hollt V (1993). The two isoforms of the mouse somatostatin receptor (mSSTR2A and mSSTR2B) differ in coupling efficiency to adenylate cyclase and in agonist-induced receptor desensitization.. FEBS Lett.

[70] Wang L, Oh DY, Bogerd J, Choi HS, Ahn RS (2001). Inhibitory activity of alternative splice variants of the bullfrog GnRH receptor-3 on wild-type receptor signaling.. Endocrinology.

[71] Surgand JS, Rodrigo J, Kellenberger E, Rognan D (2006). A chemogenomic analysis of the transmembrane binding cavity of human G-protein-coupled receptors.. Proteins.

[72] Gloriam DE, Foord SM, Blaney FE, Garland SL (2009). Definition of the G protein-coupled receptor transmembrane bundle binding pocket and calculation of receptor similarities for drug design.. J Med Chem.

[73] Gu X (2001). Maximum-likelihood approach for gene family evolution under functional divergence.. Mol Biol Evol.

[74] Gu X (2006). A simple statistical method for estimating type-II (cluster-specific) functional divergence of protein sequences.. Mol Biol Evol.

[75] Bichet DG, Birnbaumer M, Lonergan M, Arthus MF, Rosenthal W (1994). Nature and recurrence of AVPR2 mutations in X-linked nephrogenic diabetes insipidus.. Am J Hum Genet.

[76] Chini B, Mouillac B, Ala Y, Balestre MN, Trumpp-Kallmeyer S (1995). Tyr115 is the key residue for determining agonist selectivity in the V1a vasopressin receptor.. EMBO J.

[77] Postina R, Kojro E, Fahrenholz F (1996). Separate agonist and peptide antagonist binding sites of the oxytocin receptor defined by their transfer into the V2 vasopressin receptor.. J Biol Chem.

[78] Cotte N, Balestre MN, Phalipou S, Hibert M, Manning M (1998). Identification of residues responsible for the selective binding of peptide antagonists and agonists in the V2 vasopressin receptor.. J Biol Chem.

[79] Ufer E, Postina R, Gorbulev V, Fahrenholz F (1995). An extracellular residue determines the agonist specificity of V2 vasopressin receptors.. FEBS Lett.

[80] Erlenbach I, Wess J (1998). Molecular basis of V2 vasopressin receptor/Gs coupling selectivity.. J Biol Chem.

[81] Bokoch MP, Zou Y, Rasmussen SG, Liu CW, Nygaard R (2010). Ligand-specific regulation of the extracellular surface of a G-protein-coupled receptor.. Nature.

[82] Schertler GF (2008). Signal transduction: the rhodopsin story continued.. Nature.

[83] Ahuja S, Smith SO (2009). Multiple switches in G protein-coupled receptor activation.. Trends Pharmacol Sci.

[84] Hofmann KP, Scheerer P, Hildebrand PW, Choe HW, Park JH (2009). A G protein-coupled receptor at work: the rhodopsin model.. Trends Biochem Sci.

[85] Scheerer P, Heck M, Goede A, Park JH, Choe HW (2009). Structural and kinetic modeling of an activating helix switch in the rhodopsin-transducin interface.. Proc Natl Acad Sci U S A.

