# Supplementary figures and images for: Molecular Evolution of the Neuropeptide S Receptor
Source: PLoS One. 2012 Mar 30;7(3):e34046. doi: 10.1371/journal.pone.0034046 (PMC3316597; doi:10.1371/journal.pone.0034046)

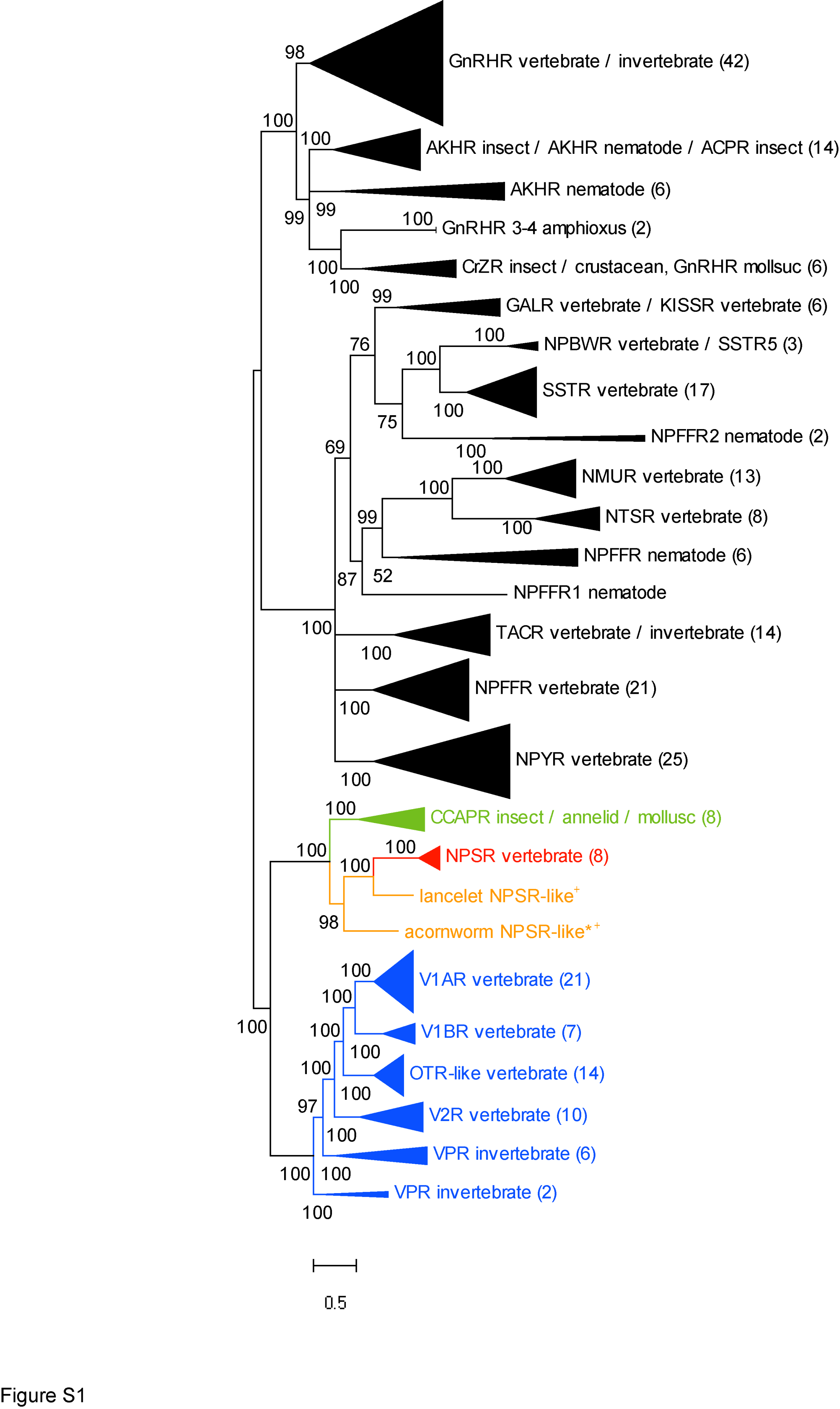

Supplement: Figure S1 — Phylogenetic relationship of the NPSR and peptide receptors. Bayesian tree of NPSR (red), invertebrate NPSR-like receptor (orange), CCAPR (green), V1AR (blue), V1BR (blue), OTR (blue), V2R (blue), VPR (invertebrate vasopressin-like receptor) (blue), the vertebrate and invertebrate GnRHR (gonadotropin releasing hormone receptor), NPFFR (neuropeptide FF receptor), TACR (tachykinin receptor), the vertebrate NMUR (neuromedin U receptor), NTSR (neurotensin receptor), GALR (galanin receptor), KISSR (kisspeptin receptor), NPBWR (neuropeptide W/neuropeptide B receptor), SSTR (somatostatin receptor), NPYR (neuropeptide Y receptor) and the invertebrate AKHR (adipokinetic hormone receptor), ACPR (adipokinetic hormone receptor/corazonin-related peptide receptors), CrzR (corazonin receptor). The tree was generated using the Bayesian approach in MrBayes 3.1.2 using JTT+F+I+G model. Analysis was run for 3000000 generations and every hundredth tree was sampled, until the average standard deviation of split frequencies dropped below the stop value of 0.02. Bayesian posterior probabilities are marked near branches as a percentage and are used as confidence values of tree branches. Nodes were compressed to represent the animal lineages. Scale bar represents the number of estimated changes per site for a unit of branch length. The receptor group abbreviations, names and accession numbers of the sequences and common and binomial names of the species are as listed in Table S2. The sequence names marked with * and +symbols represent sequences manually corrected at the N terminus and C terminus, respectively. (TIF) [file pone.0034046.s001.tif]

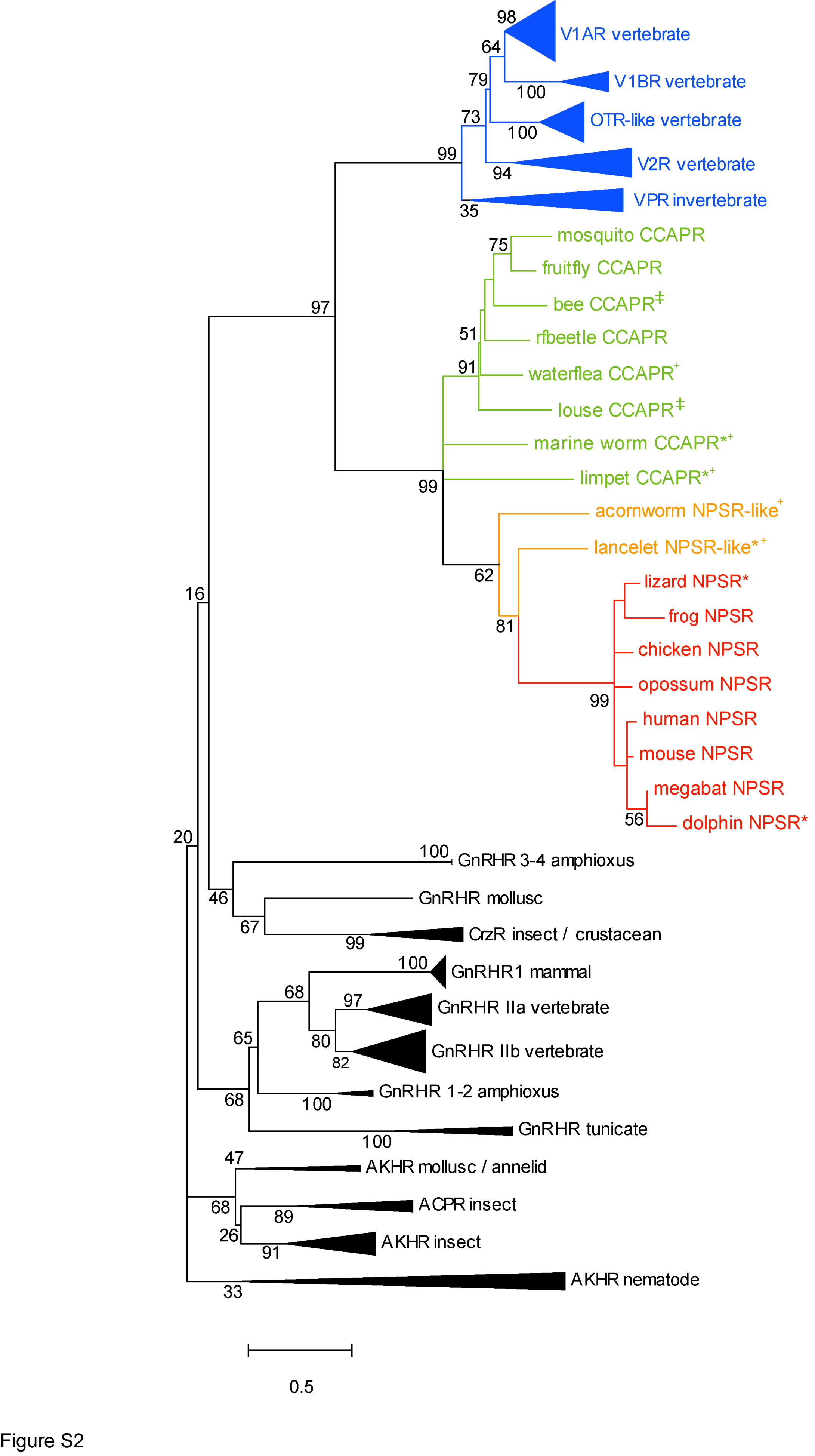

Supplement: Figure S2 — Phylogenetic relationship of the NPSR, CCAPR, GnRHR and vasopressin-like receptors from vertebrates and invertebrates. Maximum likelihood tree of NPSR (red), invertebrate NPSR-like receptor (orange), CCAPR (green), V1AR (blue), V1BR (blue), OTR (blue), V2R (blue), GnRHR (vertebrate and invertebrate Gonadotropin releasing hormone receptor) and VPR (invertebrate vasopressin-like receptor) (blue) sequences. The tree was generated using the maximum likelihood method in MEGA5.0 and bootstrap test was carried out with 500 replicates Best-fit model (JTT+I+G+F) was selected by ProtTest v2.4. Numbers on the nodes represents the bootstrap values and the clades possessing less than 50% bootstrap support were not marked. Nodes were compressed to represent the animal lineages. Scale bar represents the number of estimated changes per site for a unit of branch length. The receptor group abbreviations, names and accession numbers of the sequences and common and binomial names of the species are as listed in Table S2. The sequence names marked with * and +symbols represent sequences fragmented at the N terminus and C terminus, respectively. Sequence names marked with ‡ symbol in this figure represent fragmented sequences. (TIF) [file pone.0034046.s002.tif]

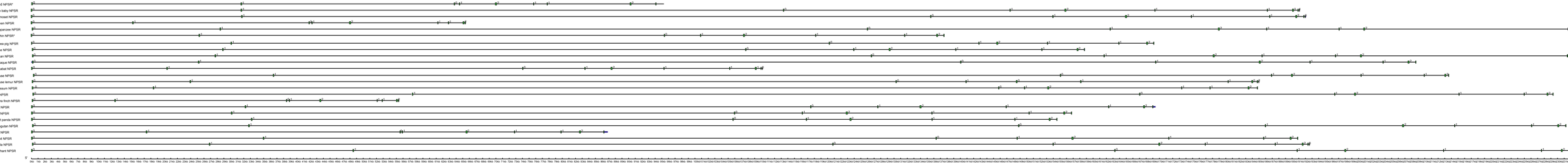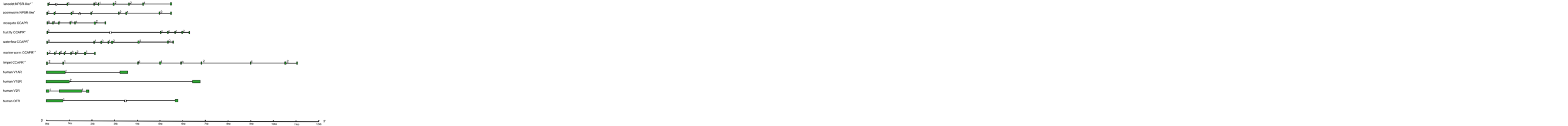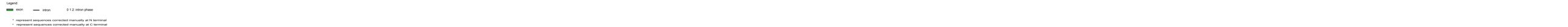

Figure S4

Supplement: Figure S4 — Gene structure representation of NPSR, NPSR-like and representative CCAPR, V1AR, V1BR, V2R and OTR sequences. (PDF) [file pone.0034046.s004.pdf]
