# Supplementary material for: Molecular Evolution of the Neuropeptide S Receptor
Source: PLoS One. 2012 Mar 30;7(3):e34046. doi: 10.1371/journal.pone.0034046 (PMC3316597; doi:10.1371/journal.pone.0034046)
Supplement: Figure S3 — Multiple sequence alignment of NPSR, NPSR-like and representative CCAPR sequences. (PDF) [file pone.0034046.s003.pdf]

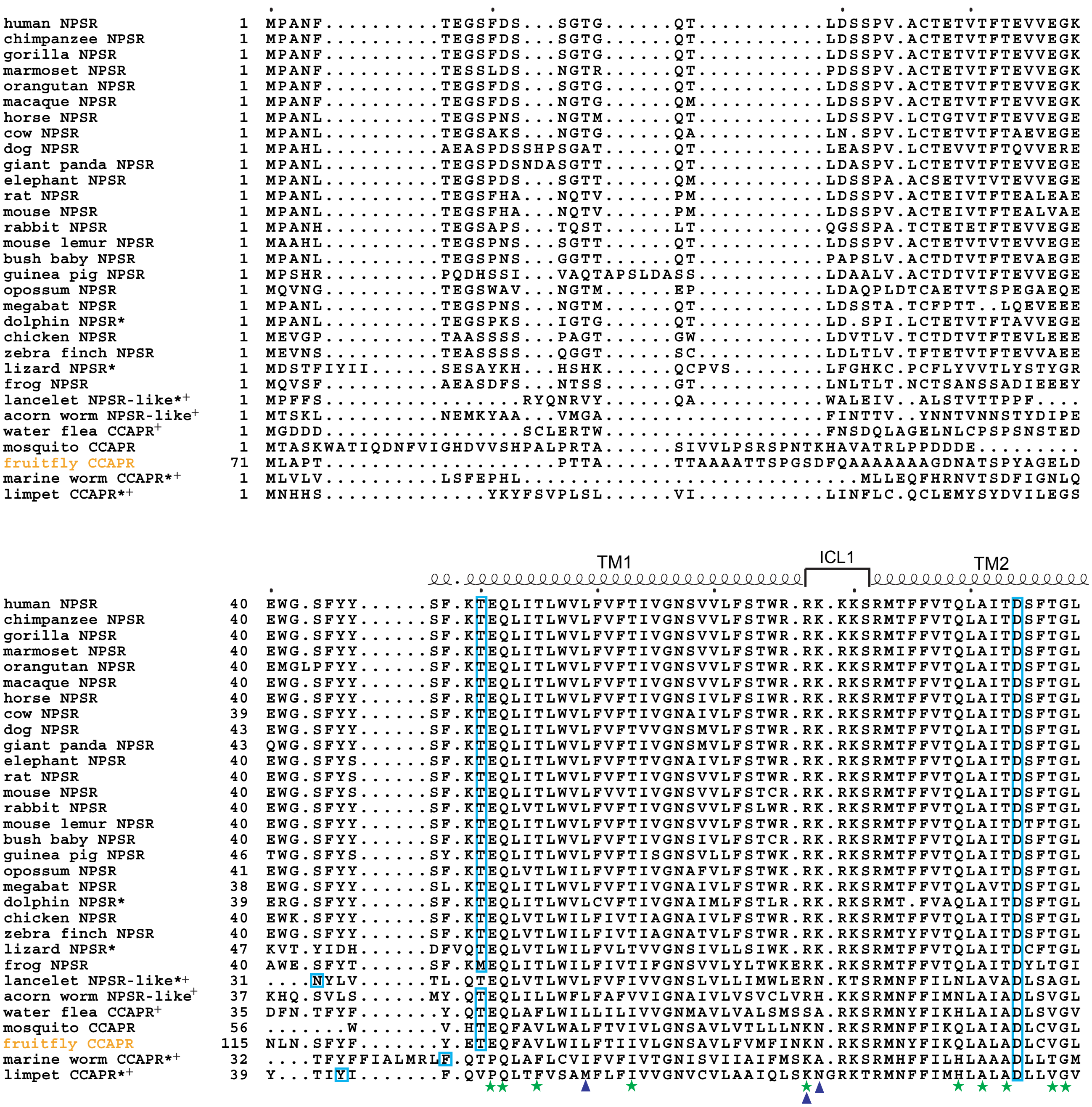

human NPSR 1 MPANF.........TEGSFDS...SGTG.....QT..........LDSSPV.ACTETVTFTEVVEGK
chimpanzee NPSR 1 MPANF.........TEGSFDS...SGTG.....QT..........LDSSPV.ACTETVTFTEVVEGK
gorilla NPSR 1 MPANF.........TEGSFDS...SGTG.....QT..........LDSSPV.ACTETVTFTEVVEGK
marmoset NPSR 1 MPANF.........TESSLDS...NGTR.....QT..........PDSSPV.ACTETVTFTEVVEGK
orangutan NPSR 1 MPANF.........TEGSFDS...SGTG.....QT..........LDSSPV.ACTETVTFTEVVEGK
macaque NPSR 1 MPANF.........TEGSFDS...NGTG.....QM..........LDSSPV.ACTETVTFTEVVEGK
horse NPSR 1 MPANL.........TEGSPNS...NGTM.....QT..........LDSSPV.LCTGTVTFTEVVEGE
cow NPSR 1 MPANL.........TEGSAKS...NGTG.....QA..........LN.SPV.LCTETVTFTAEVEGE
dog NPSR 1 MPAHL.........AEASPDSSHPSGAT.....QT..........LEASPV.LCTEVVTFTQVVERE
giant panda NPSR 1 MPANL.........TEGSPDSNDASGTT.....QT..........LDASPV.LCTETVTFTEVVEGE
elephant NPSR 1 MPANL.........TEGSPDS...SGTT.....QM..........LDSSPA.ACSETVTVTEVVEGE
rat NPSR 1 MPANL.........TEGSFHA...NQTV.....PM..........LDSSPV.ACTEIVTFTEALEAE
mouse NPSR 1 MPANL.........TEGSFHA...NQTV.....PM..........LDSSPV.ACTEIVTFTEALVAE
rabbit NPSR 1 MPANH.........TEGSAPS...TQST.....LT..........QGSSPA.TCTETETFTEVVEGE
mouse lemur NPSR 1 MAAHL.........TEGSPNS...SGTT.....QT..........LDSSPV.ACTETVTVTEVVEGE
bush baby NPSR 1 MPANL.........TEGSPNS...GGTT.....QT..........PAPSLV.ACTDTVTFTEVAEGE
guinea pig NPSR 1 MPSHR.........PQDHSSI...VAQTAPSLDASS..........LDAALV.ACTDTVTFTEVVEGE
opossum NPSR 1 MQVNG.........TEGSWAV...NGTM.....EP..........LDAQPLDTCAETVTSPEGAEQE
megabat NPSR 1 MPANL.........TEGSPNS...NGTM.....QT..........LDSSTA.TCFPTT..LQEVEEE
dolphin NPSR* 1 MPANL.........TEGSPKS...IGTG.....QT..........LD.SPI.LCTETVTFTAVVEGE
chicken NPSR 1 MEVGP.........TAASSSS...PAGT.....GW..........LDVTLV.TCTDTVTFTEVLEEE
zebra finch NPSR 1 MEVNS.........TEASSSS...QGGT.....SC..........LDLTLV.TFTETVTFTEVVAEE
lizard NPSR* 1 MDSTFIYII.....SESAYKH...HSHK.....QCPVS.......LFGHKC.PCFLYVVTLYSTYGR
frog NPSR 1 MQVSF.........AEASDFS...NTSS.....GT..........LNLTLT.NCTSANSSADIEEEY
lancelet NPSR-like*+ 1 MPFFS...............RYQNRVY.....QA..........WALEIV..ALSTVTTPPF....
acorn worm NPSR-like+ 1 MTSKL.........NEMKYAA...VMGA................FINTTV..YNNTVNNSTYDIPE
water flea CCAPR+ 1 MGDDD................SCLERTW................FNSDQLAGELNLCPSPSNSTED
mosquito CCAPR 1 MTASKWATIQDNFVIGHDVVSHPALPRTA.....SIVVLPSRSPNTKHAVATRLPPDDDE.........
fruitfly CCAPR 71 MLAPT...................PTTA.....TTAAAATTSPGSDFQAAAAAAAGDNATSPYAGELD
marine worm CCAPR*+ 1 MLVLV.........LSFEPHL............................MLLEQFHRNVTSDFIGNLQ
limpet CCAPR*+ 1 MNHHS.............YKYFSVPLSL.....VI..........LINFLC.QCLEMYSYDVILEGS
TM1
ICL1
TM2
human NPSR 40 EWG.SFYY......SF.KTEQLITLWVLFVFTIVGNSVVLFSTWR.RK.KKSRMTFFVTQLAITDSFTGL
chimpanzee NPSR 40 EWG.SFYY......SF.KTEQLITLWVLFVFTIVGNSVVLFSTWR.RK.KKSRMTFFVTQLAITDSFTGL
gorilla NPSR 40 EWG.SFYY......SF.KTEQLITLWVLFVFTIVGNSVVLFSTWR.RK.KKSRMTFFVTQLAITDSFTGL
marmoset NPSR 40 EWG.SFYY......SF.KTEQLITLWVLFVFTIVGNSVVLFSTWR.RK.RKSRMIFFVTQLAITDSFTGL
orangutan NPSR 40 EMGLPFYY......SF.KTEQLITLWVLFVFTIVGNSVVLFSTWR.RK.KKSRMTFFVTQLAITDSFTGL
macaque NPSR 40 EWG.SFYY......SF.KTEQLITLWVLFVFTIVGNSVVLFSTWR.RK.RKSRMTFFVTQLAITDSFTGL
horse NPSR 40 EWG.SFYY......SF.RTEQLITLWVLFVFTIVGNSIVLFSTWR.RK.RKSRMTFFVTQLAITDSFTGL
cow NPSR 39 EWG.SFYY......SF.KTEQLITLWVLFVFTIVGNAIVLFSTWR.RK.RKSRMTFFVTQLAITDSFTGL
dog NPSR 43 EWG.SFYY......SF.KTEQLITLWVLFVFTVVGNSMVLFSTWR.RK.RKSRMTFFVTQLAITDSFTGL
giant panda NPSR 43 QWG.SFYY......SF.KTEQLITLWVLFVFTIVGNSIVLFSTCR.RK.RKSRMTFFVTQLAITDSFTGL
elephant NPSR 40 EWG.SFYS......SF.KTEQLITLWVLFVFTTVGNAIVLFSTWR.RK.RKSRMTFFVTQLAITDSFTGL
rat NPSR 40 EWG.SFYS......SF.KTEQLITLWVLFVFTIVGNSVVLFSTWR.RK.RKSRMTFFVTQLAITDSFTGL
mouse NPSR 40 EWG.SFYS......SF.KTEQLITLWVLFVVTIVGNSVVLFSTCR.RK.RKSRMTFFVTQLAITDSFTGL
rabbit NPSR 40 EWG.SFYY......SF.KTEQLVTLWVLFVFTIVGNSVVLFSLWR.RK.RKSRMTFFVTQLAITDSFTGL
mouse lemur NPSR 40 EWG.SFYY......SF.KTEQLITLWVLFVFTIVGNSVVLFSTWR.RK.RKSRMTFFVTQLAITDTFTGL
bush baby NPSR 40 EWG.SFYY......SF.KTEQLITLWVLFVFTIVGNSIVLFSTCR.RK.RKSRMTFFVTQLAITDSFTGL
guinea pig NPSR 46 TWG.SFYS......SY.KTEQLITLWVLFVFTISGNSVLLFSTWK.RK.RKSRMTFFVTQLAITDSFTGL
opossum NPSR 41 EWG.SFYY......SF.KTEQLVTLWILFVFTIVGNAFVLFSTWK.RK.RKSRMTFFVTQLAITDSFTGL
megabat NPSR 38 EWG.SFYY......SL.KTEQLITLWVLFVFTIVGNAIVLFSTWR.RK.RKSRMTFFVTQLAVTDSFTGL
dolphin NPSR* 39 ERG.SFYY......SF.KTEQLITLWVLCVFTIVGNAIMLFSTLR.RK.RKSRMT.FVAQLAITDSFTGL
chicken NPSR 40 EWK.SFYY......SF.KTEQLVTLWILFIVTIAGNAIVLFSTWR.RK.RKSRMTFFVTQLAITDSFTGL
zebra finch NPSR 40 EWG.SFYY......SF.KTEQLVTLWILFIVTIAGNATVLFSTWR.RK.RKSRMTYFVTQLAITDSFTGL
lizard NPSR* 47 KVT.YIDH......DFVQTEQLVTLWILFVLTVVGNSIVLLSIWK.RK.RKSRMTFFVTQLAITDCFTGL
frog NPSR 40 AWE.SFYT......SF.KMEQLITLWILFIVTIFGNSVVLYLTWKERK.RKSRMTFFVTQLAITDYLTGI
lancelet NPSR-like*+ 31 ....NYLV......TL.QTEQLVTLWVLFVFIVVGNSLVLLIMWLERN.KTSRMNFFILNLAVADLSAGL
acorn worm NPSR-like+ 37 KHQ.SVLS......MY.QTEQLILLWFLFAFVVIGNAIVLVSVCLVRH.KKSRMNFFIMNLAIADLSVGL
water flea CCAPR+ 35 DFN.TFYF.......Y.QTEQLAFLWILLILIVVGNMAVLVALSMSSA.RKSRMNYFIKHLAIADLSVGV
mosquito CCAPR 56 .......W.......V.HTEQFAVLWALFTVIVLGNSAVLVTLLLNKN.RKSRMNFFIKQLAIADLCVGL
fruitfly CCAPR 115 NLN.SFYF.......Y.ETEQFAVLWILFTIIVLGNSAVLFVMFINKN.RKSRMNYFIKQLALADLCVGL
marine worm CCAPR*+ 32 ....TFYFFIALMRLF.QTPQLAFLCVIFVFIVTGNISVIIAIFMSKA.RKSRMHFFILHLAAADLLTGM
limpet CCAPR*+ 39 Y...TIYI.......F.QVPQLTFVSAMFLFIVVGNVCVLAAIQLSKNGRKTRMNFFIMHLALADLLVGV

```
                          2.57 TM2 2.61 2.63 2.65 2.68 ECL1          TM3                               ICL2          TM4
human NPSR             100  VNILTDINWRFTGDFTAPDLVCRVVRYLQVVLLYASTYVLVSLSIDRYHAIVYPMKFLQG.EKQARVLIV
chimpanzee NPSR        100  VNILTDIIWRFTGDFMAPDLVCRVVRYLQVVLLYASTYVLVSLSIDRYHAIVYPMKFLQG.EKQARVLIV
gorilla NPSR           100  VNILTDIIWRFTGDFMAPDLVCRVVRYLQVVLLYASTYVLVSLSIDRYHAIVYPMKFLQG.EKQARVLIV
marmoset NPSR          100  VNILTDIIWRFTGDFVAPDLVCRVVRYLQVVLLYASTYVLVSLSIDRYHAIVYPMKFLQG.EKQARVLIV
orangutan NPSR         101  VNILTDIIWRFTGDFMAPDLVCRVVRYLQVVLLYASTYVLVSLSIDRYHAIVYPMKFLQG.EKQARVLIV
macaque NPSR           100  VNILTDIIWRFTGDFMAPDLVCRVVRYLQVVLLYASTYVLVSLSIDRYHAIVYPMKFLQG.EKQAKVLIV
horse NPSR             100  VNILTDIIWRFTGDFMAPDLVCRVVRYLQVVLLYASTYVLVSLSIDRYHAIVYPMKFLQG.EKQARVLIV
cow NPSR                99  VNILTDIIWRFTGDFMAPDLVCRVVRYLQVVLLYASTYVLVSLSIDRYHAIVYPMKFLQG.EKQAKVLIM
dog NPSR               103  VNILTDIIWRFTGDFMAPDLVCRVVRYLQVVLLYASTYVLVSLSIDRYHAIVYPMKFLQG.EKQAKVLVM
giant panda NPSR       103  INILTDIIWRFTGDFLAPDLVCRVVRYLQVVLLYASTYVLVSLSIDRYHAIVYPMKFLQG.EKQAKVLIV
elephant NPSR          100  FNILTDIIWRFTGDFMAPDLVCRVVRYFQVVLLYASTYVLVSLSIDRYHAIVYPMKFLQG.EKQAKVLIV
rat NPSR               100  INILTDIIWRFTGDFMAPDLVCRIVRYLQVVLLYASTYVLVSLSIDRYHAIVYPMKFLQGAEKQAKVLIG
mouse NPSR             100  INILTDIIWRFTGDFMAPDLVCRVVRYLQVVLLYASTYVLVSLSIDRYHAIVYPMKFLQG.EKQAKVLIG
rabbit NPSR            100  VNILTDIIWRFTGDFMAPDLVCRVVRYLQVVLLYASTYVLVSLSIDRYHAIVHPMKFLQG.EKQAKVLIG
mouse lemur NPSR       100  VNILTDIIWRFTGDFMAPDLVCRVVRYLQVVLLYASTYVLVSLSIDRYHAIVYPMKFLQG.EKQAKVLIM
bush baby NPSR         100  VNILTDIIWRFTGDFMAPDLVCRVVRYLQVVLLYASTYVLVSLSIDRYHAIVYPMKFPQG.VKQAKVLII
guinea pig NPSR        106  VNILTDIIWRFTGDFLAPDLVCRVVRYLQVVLLYASTYVLVSLSIDRYHAIVYPMKFLQG.ERQAKVLIG
opossum NPSR           101  INILTDIIWRFTGDFMAPDLICRVIRYLQVVLLYASTYVLVSLSIDRYHAIVYPMKFLQG.EKQAKVLIV
megabat NPSR            98  VNILTDIIWRFTGDFLAPDLVCRVVRYLQVVLLYASTYVLVSLSIDRYHAIVYPMNFLQG.EKQAKILTV
dolphin NPSR*           98  VNILTDIIGRFTGDFMAPDLVC.VVHYVQVVLLYASTYVLGSLRIDRYHAIIYPMKFLQG.EKQAKVLSV
chicken NPSR           100  INIMTDIIWRYTGDFMAPDIVCRVVRYLQVVLLYASTYVLVSLSIDRYHAIVYPMKFMQG.ERQAKVLIG
zebra finch NPSR       100  INIMTDIIWRYTGDFMAPDIVCRVVRYFQVVLLYASTYVLVSLSIDRYHAIVYPMKFLQG.ERQAKVLIG
lizard NPSR*           108  INIMTDIIWRFTGDFMAPDLVCRIVRYFQVVLLYASTYVLVSLSIDRYHAIVYPMKFFQG.ERQAKILIV
frog NPSR              101  ISISVNIIWRFTGEFMAPEIVCKTVRYLQVVLLYASTYVLVSLSIDRYHAIVHPMKFLQG.EKQAKVLIA
lancelet NPSR-like*+    89  FNVLPDIVHRFVVEWIAGNTLCKLVKYTQAVLLYASTYVLVAMSIDRYDAIVHPLQFVR..EHKSKVMIS
acorn worm NPSR-like+   98  LNILPDIIHRYTREFYGGEIVCKLVKYVQAIVVYGSTYQLVALSIDRYDAIVHPMNFSG..NKRSMIMVI
water flea CCAPR+       95  ISVLTDIVWKITVAWHAGNIACKVIRFSQVLVTYSSTYVLVALSIDRYDAICHPMNFSRG.WRRARILVS
mosquito CCAPR         110  LSVLTDIIWRMTVSWKAGNAACKAIRFAQASVTYASTYVLVALSIDRYDAITHPMNFSGC.WNRARRLVA
fruitfly CCAPR         175  LNVLTDIIWRITISWRAGNVACKVIRFSQVCVTYSSTYVLVAMSIDRYDAITHPMNFSKS.WKRARHLVA
marine worm CCAPR*+     96  ITVVTDLSWKITVEWHAGLFGCKVIRFLQSVVVFAANYILVALSIDRLDAIARPMNFSSS.GRRAKMLVS
limpet CCAPR*+          98  ICVMTDLLSKITIEWHAGNAMCKILQYLQATVTYLSTFVLVSLSIDRYDAVARPMNFSRS.AYQARLLIT

                                 TM4                ECL2                     TM5        5.58   ICL3
human NPSR             169  IAWSLSFLFSIPTLIIFGKRTLSNGEVQCWALWPDDSYWTPYMTIVAFLVYFIPLTIISIMYGIVIRTIW
chimpanzee NPSR        169  IAWSLSFLFSIPTLIIFGKRTLSNGEVQCWALWPDDSYWTPYMTIVAFLVYFIPLTIISIMYGIVIRTIW
gorilla NPSR           169  IAWSLSFLFSIPTLIIFGKRTLSNGEVQCWALWPDDSYWTPYMTIVAFLVYFIPLTIISIMYGIVIRTIW
marmoset NPSR          169  IAWGLSFLFSIPTLIIFGKRTLSNGEVQCWALWPDDSYWTPYMTVVAFLVYFIPLTIISVMYGIVIRTIW
orangutan NPSR         170  IAWTLSFLFSVPTLIIFGKRTLSNGEVQCWALWPDDSYWTPYMTIVAFLVYFIPLTIISIMYGIVIRTIW
macaque NPSR           169  IAWSLSFLFSIPTLIIFGKRTLSNGEVQCWALWPDDSYWTPYMTIVAFLVYFIPLTIISVMYGIVIRTIW
horse NPSR             169  IAWGLSFLFSIPTLIIFGKRTLSNGEVQCWALWPDDSYWTPYMTIVAFLVYFIPLTIISVIYAIVIRTIW
cow NPSR               168  IAWSLSFLFSIPTLIIFGKRKLSNGEVQCWALWPDDSYWTPYMTIVAFLVYFIPLTIISVIYAIVIRTIW
dog NPSR               172  IAWSLSFLFSIPTLIIFGKRKLPNGEVQCWALWPDDSYWTPYMTIVAFLVYFIPLTIISVIYAIVIRTIW
giant panda NPSR       172  IAWSLSFLFSIPTLIIFGKRKLPSGEVQCWALWPDDSYWTPYMTIVAFLVYFIPLTIISVIYAIVIRTIW
elephant NPSR          169  IAWSLSFLFSIPTLIIFGKRKLSNGEVQCWALWPDDSYWTPYMTIVAFLVYFIPLTIISVIYGIVIRTIW
rat NPSR               170  IAWSLSFLFSIPTLIIFGKRTLSNGEVQCWALWPDDSYWTPYMTIVAFLVYFIPLTIISVIYGLVIRTIW
mouse NPSR             169  IAWSLSFLFSIPTLIIFGKRTLSNGEVQCWALWPDDSYWTPYMTIVAFLVYFIPLAIISVIYGLVIRTIW
rabbit NPSR            169  IAWSLSFLFSIPTLIIFGKRTLSNGEVQCWALWPDDSYWTPYMTIVAFLVYFIPLTIISVIYGIVIRTIW
mouse lemur NPSR       169  IAWILAFLFSIPTLIIFGKRTLSNGEVQCWALWPDDSY.IPYMTIVXFLVYFIPLTIISVIYGIVIRTIW
bush baby NPSR         169  IAWILSFLFSIPTLIIFGKRTLSNGEVQCWALWPDDSYWTPYMTIVAFLVYFIPLTIISVIYGIVIRTIW
guinea pig NPSR        175  VAWSLSFLFSIPTLIIFGKRTLSNGEVQCWALWPDDSYWTPYMTVVAVLVYFIPLTILSIIYSIVIRTIW
opossum NPSR           170  VAWSLSFLFSIPTLIIFGKRKLANGEVQCWALWPDDSYWIPYMTIVAFLVYFIPLIIISVIYFIVIRTIW
megabat NPSR           167  TAWSLSFLFSIPTLIIFGKRKLSNGEVQCWALWPDDSYWTPYMTIVAFLVYFIPLAIISVIYAIVIQTIW
dolphin NPSR*          166  IAWSFSFLFSIPTLIIFGKRKLSNGEVQCWAL.PDDSYQTPHMTIVAFLVYFIPLTIISVIYAIVIRMIW
chicken NPSR           169  VAWSLSFLFSIPTLIIFGKRQLSNGEVQCWALWPDDSYWIPYMTVVAFLVYFIPLIIISVIYSIVIRTIW
zebra finch NPSR       169  VAWSLSFLFSIPTLIIFGKRQLSNGEVQCWALWPDESYWIPYMTVVAFLVYFIPLIIISVIYSIVIRTIW
lizard NPSR*           177  IAWGLSFLFSIPTLIIFGKRQLSNGEVQCWAVWPDDSYWIPYMTTVAFLVYFIPLIIISVIYFIVIRTIW
frog NPSR              170  VSWTLSFLFSIPTFIIFGKLKLPNGEMQCWALWPDDSYWTPYMTIVALLVYFIPLIIISVIYFIVIRTIW
lancelet NPSR-like*+   157  VAWGLALLFSVPSPVIFAVRRQPNGEWQCWAEWPEDWYWVPYMTVVAAFVFFIPLGIISICYAIIVKIW
acorn worm NPSR-like+  166  SMWVVAFILAVPSPVFFEETVLENGEVQCWIELPQTWVWKPYSVILAFLLFFIPLVIVTFCYSVIIYTIW
water flea CCAPR+      164  VAWILSAIFSSPMLVLYEQG.LVQGQVQCWIDFTAPWQWQLYLTLVAVSLLVLPALLIFACYIVIVRTIW
mosquito CCAPR         179  AAWSLSALFSLPIFYLYEER.SIQGKKQCWIDLVDPWRWQVYMCWVASSLFVLPALIISACYAIIVKTIW
fruitfly CCAPR         244  GAWVVSALFSLPILVLYEEK.LIQGHPQCWIELGSPMAWQIYMCLVSATLFAVPALIISACYAIIVKTIW
marine worm CCAPR*+    165  TAWILAFLFSAPMGIIPELT.VRRGLVQCFISLPEPWHWKVYITILAFALFILPAIIISTCYIIIVIVIW
limpet CCAPR*          167  FAWLSALMFALPAPFLYHEE.EQQGKIQCWMDFPEYWHWQLFFTLVSVVTFILPAIIISGCYIAIISIIW
```

```
                                                              ICL3                              TM6        6.49
human NPSR            239  IKSKTY.ETVI....SN.........CSDGK.....LCSSY.NRGLISKAKIKAIKYSIIIILAFICCWS
chimpanzee NPSR       239  IKSKTY.ETVI....SN.........CSDGK.....LCSSY.NRGLISKAKIKAIKYSIIIILAFVCCWS
gorilla NPSR          239  IKSKTY.ETVI....SN.........CSDGK.....LCSSY.NRGLISKAKIKAIKYSIIIILAFICCWS
marmoset NPSR         239  IKSKTY.ESVI....SN.........CSDGK.....LCSRY.NRGPISKAKLKAIKYSIVIILAFICCWS
orangutan NPSR        240  IKSKTY.ETVI....SN.........CSDGK.....LCSSY.NRGLISKAKIKAIKYSIIIILAFICCWS
macaque NPSR          239  IKSKTY.ETVI....SN.........CSDGK.....LCSSY.NRGLISKAKIKAIKYSIVIILAFICCWS
horse NPSR            239  TKSKAS.ETVI....SN.........CSDGK.....LCTSY.NRGLISKAKIKAIKYSIVIILAFICCWS
cow NPSR              238  VKSKAY.ETVI....SN.........CSDGK.....LCTSY.NRGLISKAKIKAIKYSIVIILAFICCWS
dog NPSR              242  IKSKAH.ETVI....SN.........CSGGK.....LCTSY.NRGLISKAKIKAIKYSIVIILAFICCWS
giant panda NPSR      242  IKSKAH.ETVI....SN.........CLDGK.....LCTSY.HRGLISKAKIKAIKYSIVIILAFICCWS
elephant NPSR         239  IKSKAH.DMVI....SN.........CSNGK.....LCTSY.NRRLISKAKIKAIKYSVVIILAFICCWS
rat NPSR              240  IKSKAH.ETVI....SN.........CSDGE.....LCCSY.NRGLISKAKIKAIKYSIVIILAFICCWS
mouse NPSR            239  MKSKTH.ETVI....SN.........CSDGK.....LCCSY.NRGLISKAKIKAIKYSIVIILAFICCWS
rabbit NPSR           239  VKSKAC.ETVN....SN.........CSDGK.....LCTSY.NRGLISKAKIKAVKYSAAIILAFICCWS
mouse lemur NPSR      238  IKSKAH.ETVI....SN.........CSGGK.....LCTSY.NRGLISKAKIKAIKYSIVIILAFICCWS
bush baby NPSR        239  IKSKAH.DSVI....SN.........CSGGK.....LCTSY.NRGLISKAKIKAIKYSIVIILAFICCWS
guinea pig NPSR       245  MKSKAP.ETVI....SN.........YSDGK.....LCTSY.NRGFISKAKVKAIKYSIVIVLAFTICWS
opossum NPSR          240  IKSKAH.DMII....SN.........CSEGK.....LCSSY.NRGVISKAKIKAIKYSIVIIFAFVICWS
megabat NPSR          237  MKSKGHDESVI....SN.........CSDGK.....LCTSY.NRGLISKAKIKAIKYSMVIILAFICCWS
dolphin NPSR*         235  VKSKAH.ETEI....SS.........CSDGK.....RCTIS.N.GLISKAKINAIKYSIVIILAFICCWS
chicken NPSR          239  MKSKAQ.AVIV....SS.........CTDGK.....TSAGYTSRGFISRAKVKAIKYSIVIVLAFALCWS
zebra finch NPSR      239  MKSKAQ.AAII....SS.........CPGGK.....TSRGYTSRGFISRAKVKAIKYSIVIILAFILCWS
lizard NPSR*          247  TKSKAH.AIIV....SN.........CSDGK.....FCTSYTHRGLISKAKIKAVKYSAVIILAFVLCWS
frog NPSR             240  VKSKGH.AVII....SN.........YTDGN.....FCTSYSHRGLISKAKMKAIKYSIVVILAFILCWS
lancelet NPSR-like*+  227  KRGKGM.AYEDHIPRSR.........ASSGKNFYRIFHLIHINNGFTSRAKARTIKLSVAIILAFICCWS
acorn worm NPSR-like+ 236  RKSKMM.VPAK....RRIFN......EKNGD...........SRGLIPKAKIKTIKMTLCIVLSFIVCWS
water flea CCAPR+     233  VQSAIF.LGIN....RAGGSAGTAMLDEDQE.....SRRA.SSRGIIPRAKIKTVKMTFVIVFVFILCWA
mosquito CCAPR        248  AKGAIM.GPID....RTRNGMA......DLA.....SRRA.SSRGIIPKAKVKTVKMTIVIVIVFVLCWS
fruitfly CCAPR        313  AKGSIF.VPTD....RA..GFG......GAA.....TRRA.SSRGIIPRAKVKTVKMTLTIVFVFIICWS
marine worm CCAPR*+  234  SKGNVS.TTSN....KR.......QLSKRGR.....ESTT.SSRGMIPKARIKTIKMTFAIVTVFIICWV
limpet CCAPR*+       236  SKGQSRRDGMD....ETHNLPR....IGYGSREHMVCRRTNSNRGIIPQAKIRTIKMTLIIVIVFILCWS

                              TM6      ECL3   6.70          TM7               Helix 8
human NPSR            289  PYFLFDILDNFNLLPDTQERFYASVIIQNLPALNSAINPLIYCVFSSSISFPCRV..............
chimpanzee NPSR       289  PYFLFDILDNFNLLPDTQERFYASVIIQNLPALNSAINPLIYCVFSSSISFPCRV..............
gorilla NPSR          289  PYFLFDILDNFNLLPDTQERFYASVIIQNLPALNSAINPLIYCVFSSSISFPCRA..............
marmoset NPSR         289  PYFLFDILDNFNLLPDTQERFYASVIIQNLPALNSAMNPLIYCVFSSSISFPCRA..............
orangutan NPSR        290  PYFLFDILDNFNLLPDTQERFHASVIIQNLPALNSAINPLIYCVFSSSISFPCRE..............
macaque NPSR          289  PYFLFDILDNFNLLPDTQERFYASVIIQNLPALNSAINPLIYCVFSSSISFPCGE..............
horse NPSR            289  PYFLFDILDNFSLLPDTKERFYASVIIQNLPALNSAINPLIYCVFSSSICFPCGD..............
cow NPSR              288  PYFLFDILDNFSLLPNTKERFYASVIIQNLPALNSAINPLIYCVFSNSICFPCRE..............
dog NPSR              292  PYFLFDILDNFSLLPDTEERFYASVIIQNLPALNSAINPLIYCVFSSSICFPCRE..............
giant panda NPSR      292  PYFFFDILDNFRLLPDTEERFYASVIIQNLPALNSAVNPLIYCVFSSSVCFPCRE..............
elephant NPSR         289  PYFLFDILDNFSILPDTKERFYASVIIQNLPALNSAINPLIYCAFSSSICFPCRE..............
rat NPSR              290  PYFLFDMLDNFNLLPDTKERFYASVIIQNLPALNSAINPLIYCIFSGSLCSPCKV..............
mouse NPSR            289  PYFLFDILDNFNVLPDTKERFYASVIIQNLPALNSAINPLIYCIFSSSICSPCKM..............
rabbit NPSR           289  PYFLFDILDNFNFLPNTKERFYASVIIQNLPALNSAINPLIYCVFSSTVCFPFRE..............
mouse lemur NPSR      288  PYFLFDILDNFNLLPDTKERFYASVIIQNLPALNSAINPLIYCAFSSSICFPWGT..............
bush baby NPSR        289  PYFLFDILDNFSFFPDTKERFYASVIIQNLPALRR.INPLVYCAFS.SIC.PWGA..............
guinea pig NPSR       295  PYFLFDILDNFNLLPDTKERFYASVIIQNLPALNSAINPLIYCIFNRPICFPCRE..............
opossum NPSR          290  PYFLFDILDNFSLLPETKERFYASVIIQNLPALNSAINPLIYCLFSNSLCYPCRE..............
megabat NPSR          288  PYFLFDMLDNFGLLPDTEERFYASVIIQNLPALNSAINPLIYCAFSSSLCFPRRV..............
dolphin NPSR*         284  PYFLFDILDNFSLLPNTKERFYASVIIQNLPALNNAINPLIYCVFSSSICFPCGA..............
chicken NPSR          290  PYFLFDILDNFNILPETKERFYASVIIQNLPALNSAINPLIYCLFSNHLCTPFEE..............
zebra finch NPSR      290  PYFLFDILDNFNILPETKERFYASVIIQNLPALNSAVNPLIYCFFSNSLCPPSEE..............
lizard NPSR*          298  PYFLFDILDNFSILPETKERFYAAVIIQNLPALNSAINPIIYCIFSDTLCQAFRS..............
frog NPSR             291  PYFLFDILDNFEMLPETKERFYASVIIQHLPFLNSAINPIIYCVFSNRHCRLSRD..............
lancelet NPSR-like*+  287  PYFLFDILDNYDALPDTQAKKEASLIIQNLPALNSAINPIIYGFFSTKLYRKLRG..............
acorn worm NPSR-like+ 284  PFTLWFILEIYGHIPKNDLTMTIHIIVQNLPSLNSATNPAIYGLFSTNICKELRC..............
water flea CCAPR+     292  PYIVFDLLQVYGHIPKSKTMIAVATFIQSLAPLNSAANPLIYCLFSTHLCRNLRK..............
mosquito CCAPR        301  PYIVFDLLQVFGQIPETQTNIAVATFIQSLAPLNSAANPLIYCLFSTQVCRMIKRLPPFRWLLASKWCCK
fruitfly CCAPR        364  PYIIFDLLQVFGQIPHSQTNIAIATFIQSLAPLNSAANPLIYCLFSSQVFRTLSRFPPFKWLTC...CCK
marine worm CCAPR*+  286  PFFVFDLADVYGLIPRTKHKRAIAIFIQSLATLNSATNPVIYFIFSKTQCSPLRA..............
limpet CCAPR*+       298  PFFIYNILELYETIPINDP...LSTFIQSAAPLNSAANPIIYGIFSTRICVNLRI..............
```

```
                                       HELIX 8
                          ......................ℓ
                                                             .         .
human NPSR               344  ......................IRLRQL.......QEAALMLCPQR.....E.NWK..........GTWP
chimpanzee NPSR          344  ......................IPLRQL.......QEAALTLCPQR.....Q.NWK..........GTWP
gorilla NPSR             344  .........................NSSVYLLACDVSVLWALVPTSEKESCE.SWRRKGAKITG..FQND
marmoset NPSR            344  .........................SSTVYLLACDVAVLWGLVLPGGKENCE.SLRRKGAKITG..FQND
orangutan NPSR           345  .........................RRS.......QDSRMTFRERT.....E.RHE..........MQIL
macaque NPSR             344  .........................RRS.......QDSRMTFRERT.....E.RHE..........MQIL
horse NPSR               344  .........................QRS.......QDSRMTYQERT.....E.RHE..........MQVL
cow NPSR                 343  .........................QRS.......RDSRMTCRERT.....E.KHE..........MQVL
dog NPSR                 347  .........................QTS.......RGSRKTFRERT.....Q.RHE..........MQVL
giant panda NPSR         347  .........................RTS.......RDSRMTFRERT.....Q.RHE..........MQVL
elephant NPSR            344  .........................RKS.......RDSRMTCQERT.....E.RHE..........MQVL
rat NPSR                 345  .........................QRS.......QDSRMTYRERS.....E.RHE..........MQIL
mouse NPSR               344  .........................QRS.......QDSRMTYRERS.....E.RHE..........MQIL
rabbit NPSR              344  .........................RRS.......QDSRMTCRERT.....E.RHE..........MQTL
mouse lemur NPSR         343  .........................RNSVYLLACDASSVHWAVLTSGKEGCE..WRKKGAKITG..FQSD
bush baby NPSR           341  .........................NSSAYLLAD.DASVLTWVLTSGKEGCE.RKE...AKITG..FQND
guinea pig NPSR          350  .........................QKS.......QVSRMTCRERS.....E.RHE..........LQFL
opossum NPSR             345  .........................RRN.........AGGTFQDKT.....E.RHE..........MRMR
megabat NPSR             343  .........................NSSAYLLAS.AVIHWALVLGSGGK..........AKITG..FQDD
dolphin NPSR*            339  .........................NSSAYLLASDVSVHWALVLTSRKEGCE.WWG..............
chicken NPSR             345  .........................RRT.......RRLEGTFRDRS.....DGGQE..........MQVL
zebra finch NPSR         345  .........................RRT.......QRLGGTFRERS.....DGGQQ..........MQVL
lizard NPSR*             353  .........................RRR.......SGNLGTFRERT.....E.GQE..........MQVL
frog NPSR                346  .........................RNS.......GKLGGTIRDKT.....E.GIE..........MQVV
lancelet NPSR-like*+     342  .........................RRA.......LKHFKMLKAFH.....LKNHF..........VEEG
acorn worm NPSR-like+    339  ...........................MVTVVREVLSLMTNIHNRN.....GYSNF..........RNIV
water flea CCAPR+        347  ................................ETLTAYNGIWNKTS.........IFPFHFQNGHTI...
mosquito CCAPR           371  SPDGGSQGAGMRNGTVLNGNARLQNHNSSDSMRTLTTSLTISQRSC.....IRP.SRVVIVERPKAAL..
fruitfly CCAPR           431  SYRNNSQQN..RCHTV...GRRL..HNSCDSMRTLTTSLTVSRRST.....NKANARVIICERPNKVITV
marine worm CCAPR*+      341  ............ETLSVAIRKLKLEKYKKSCTTSSSHLLFQKAPV..................LRVAFT
limpet CCAPR*+           350  ............SRLSSCDYSMTQDYESVSSVTFTIRLTRRLECT........................
                                                                ▲
```

```
                               .
human NPSR               369  GVPSWALPR
chimpanzee NPSR          369  GVPSWALPR
gorilla NPSR             386  VPGEN.REA
marmoset NPSR            386  VPGEN...R
orangutan NPSR           367  SKPEF...I
macaque NPSR             366  SKPEF...I
horse NPSR               366  SKPEF...I
cow NPSR                 365  SKPEF...M
dog NPSR                 369  SKPEF...I
giant panda NPSR         369  PKPEF...I
elephant NPSR            366  SKPEF...I
rat NPSR                 367  SKPEF...I
mouse NPSR               366  SKPEF...I
rabbit NPSR              366  AKPEF...L
mouse lemur NPSR         384  IPGED...G
bush baby NPSR           379  IPREN...R
guinea pig NPSR          372  SKPEF...I
opossum NPSR             365  PKPEF...I
megabat NPSR             375  VPREN...R
dolphin NPSR*            369  ....R...K
chicken NPSR             368  SKPEY...I
zebra finch NPSR         368  SKPEC...I
lizard NPSR*             375  SKPEY...I
frog NPSR                368  SRPEY...L
lancelet NPSR-like*+     365  SVFLC...L
acorn worm NPSR-like+    367  NRAMY...I
water flea CCAPR+        373  ..LKK...D
mosquito CCAPR           433  .AMSQ...V
fruitfly CCAPR           489  PAMSE...V
marine worm CCAPR*+      380  SLLLV...L
limpet CCAPR*+           383  ........S
```

Note: Secondary structure elements marked on top of the alignment correspond to that predicted for the human NPSR sequence. Transmembrane regions are named TM1 to TM7; extracellular and intracellular loops are named ECL1, 2, 3 and ICL1, 2, 3, respectively. The upright triangle symbol in blue corresponds to Type I divergence sites and star symbol in green corresponds to Type II divergence sites. Numbers on the left of the sequences correspond to the amino acid residue position for each sequence. Intron positions are marked in blue boxes. N-terminal region of the fruitfly CCAPR sequence has been trimmed to align with rest of the sequences. Sequences corrected manually at the N and C- termini are represented with * and + at the end of their sequence names.

Figure S3
