# Supplementary material for: Molecular Evolution of the Neuropeptide S Receptor
Source: PLoS One. 2012 Mar 30;7(3):e34046. doi: 10.1371/journal.pone.0034046 (PMC3316597; doi:10.1371/journal.pone.0034046)
Supplement: Table S1 — List of identified neuropeptide S receptors. (DOC) [file pone.0034046.s005.doc]

**Table S1.** List of identified Neuropeptide S receptors.

| **Binomial nomenclature** | **Common Name /Names used in further studies** | **Accession number** |
| --- | --- | --- |
| *Homo sapiens*^d#^ | human NPSR^p^ | ENSP00000352839^a^ |
| *Otolemur garnettii*^d#^ | bush baby NPSR | ENSOGAP00000009225^a^ |
| *Callithrix jacchus*^d#^ | marmoset NPSR | ENSCJAP00000022279^a^ |
| *Gallus gallus*^d#^ | chicken NPSR^p^ | ENSGALP00000019838^a^ |
| *Pan troglodytes*^d#^ | chimpanzee NPSR | ENSPTRP00000045319^a^ |
| *Tursiops truncatus*^d#^ | dolphin NPSR* ^p^ | ENSTTRP00000015651^a^ |
| *Cavia porcellus*^d#^ | guinea pig NPSR | XP_003470021^b^ |
| *Equus caballus*^d#^ | horse NPSR | ENSECAP00000010898^a^ |
| *Macaca mulatta*^d#^ | macaque NPSR | ENSMMUP00000035218^a^ |
| *Pteropus vampyrus*^d#^ | megabat NPSR^p^ | ENSPVAP00000016321^a^ |
| *Mus musculus*^d#^ | mouse NPSR^p^ | ENSMUSP00000056432^a^ |
| ***Microcebus murinus***^d#^ | mouse lemur NPSR | ENSMICP00000007592^a^ |
| *Rattus norvegicus*^d#^ | rat NPSR | ENSRNOP00000021625^a^ |
| *Taeniopygia guttata*^d#^ | zebra finch NPSR | ENSTGUP00000005494^a^ |
| *Monodelphis domestica*^d#^ | opossum NPSR^p^ | XP_001365641^b^ |
| *Bos taurus*^d#^ | cow NPSR | NP_001179906^b^ |
| *Canis familiaris*^d#^ | dog NPSR | XP_539511^b^ |
| *Ailuropoda melanoleuca*^d#^ | giant panda NPSR | XP_002918703^b^ |
| *Pongo abelii*^d#^ | orangutan NPSR | XP_002818110^b^ |
| ***Xenopus tropicalis***^d#^ | frog NPSR^p^ | XP_002937035^b^ |
| *Oryctolagus cuniculus*^d#^ | rabbit NPSR | NP_001191373^b^ |
| *Gorilla gorilla*^d#^ | gorilla NPSR | ENSGGOP00000011480^a^ |
| *Loxodonta africana*^d#^ | elephant NPSR | ENSLAFP00000015087^a^ |
| *Branchiostoma floridae*^#^ | lancelet NPSR-like*^+p^ | 212588 ^c^ |
| *Saccoglossus kowalevskii*^#^ | acorn worm NPSR-like^+p^ | XP_002732566^b^ |
| *Anolis carolinensis*^#^ | lizard NPSR*^p^ | XP_003222313^b^ |
| *Sus scrofa* | pig NPSR | XP_003134844^b^ |
| *Meleagris gallopavo* | turkey NPSR | XP_003207347^b^ |
| *Vicugna pacos* | alpaca NPSR ° | ENSVPAP00000009304^a^ |
| *Dasypus novemcinctus* | armadillo NPSR ° | ENSDNOP00000003020^a^ |
| *Felis catus* | cat NPSR ° | ENSFCAP00000006534^a^ |
| *Erinaceus europaeus* | hedgehog NPSR ° | ENSEEUP00000006102^a^ |
| *Procavia capensis* | hyrax NPSR ° | ENSPCAP00000004629^a^ |
| *Dipodomys ordii* | kangaroo rat NPSR ° | ENSDORP00000003505^a^ |
| *Echinops telfairi* | lesser hedgehog tenrec NPSR ° | ENSETEP00000016539^a^ |
| *Myotis lucifugus* | microbat NPSR ° | ENSMLUP00000012652^a^ |
| *Ochotona princeps* | pika NPSR ° | ENSOPRP00000006534^a^ |
| *Sorex araneus* | shrew NPSR ° | ENSSARP00000005435^a^ |
| *Choloepus hoffmanni* | sloth NPSR ° | ENSCHOP00000000687^a^ |
| *Spermophilus tridecemlineatus* | squirrel NPSR ° | ENSSTOP00000007424^a^ |
| *Tarsius syrichta* | tarsier NPSR ° | ENSTSYP00000006128^a^ |
| *Macropus eugenii* | wallaby NPSR ° | ENSMEUP00000014078^a^ |

^a^ Sequence from Ensembl database,  ^b^ Sequence from NCBI database and ^c^ Sequence from JGI database.

Sequences corrected manually at N and C- termini are represented with * and ^+^ on their sequence names

° Represents incomplete sequences.

^p^ Sequences used in the phylogeny.

^d^ Sequences used in the functional divergence analysis (Data S3).

^#^ Represents sequences used for gene structure analyses (Figure S4, Data S2).
