# Supplementary material for: Molecular Evolution of the Neuropeptide S Receptor
Source: PLoS One. 2012 Mar 30;7(3):e34046. doi: 10.1371/journal.pone.0034046 (PMC3316597; doi:10.1371/journal.pone.0034046)
Supplement: Table S2 — Accession numbers of all protein sequences used in the phylogenetic analyses. (DOC) [file pone.0034046.s006.doc]

**Table S2. Accession numbers of all protein sequences used in the phylogenetic analyses**

| **Binomial Nomenclature** | **Common Name** | **Name and receptor abbreviation used in phylogeny** | **Receptor name** | **Accession number** |
| --- | --- | --- | --- | --- |
| *Equus caballus* | Horse | V1AR | Vasopressin 1A | XP_001917958^b^ |
| *Homo sapiens* | Human | V1AR | Vasopressin 1A | ENSP00000299178^a^ |
| *Loxodonta africana* | Elephant | V1AR | Vasopressin 1A | ENSLAFP00000005037^a^ |
| *Tursiops truncatus* | Dolphin | V1AR | Vasopressin 1A | ENSTTRP00000001752^a^ |
| *Mus musculus* | Mouse | V1AR | Vasopressin 1A | ENSMUSP0000002032^a^ |
| *Monodelphis domestica* | Opossum | V1AR | Vasopressin 1A | XP_001372716^b^ |
| *Gallus gallus* | Chicken | V1AR | Vasopressin 1A | NP_001103908^b^ |
| *Anolis carolinensis* | Anole lizard | V1AR | Vasopressin 1A | XP_003221310^b^ |
| ***Xenopus tropicalis*** | Western clawed frog | V1AR | Vasotocin | XP_002931664^b^ |
| *Bufo marinus* | Marine toad | V1AR | Vasotocin | BAF48112^b^ |
| *Rana catesbeiana* | Bullfrog | V1AR | Vasotocin | AAQ22364^b^ |
| *Oryzias latipes* | Medaka | V1AR | Vasotocin | ENSORLP00000002643^a^ |
| *Platichthys flesus* | European flounder | V1AR | Vasotocin | AAF00506^b^ |
| *Paralichthys olivaceus* | Japanese flounder | V1AR | Vasotocin | ABO38815^b^ |
| *Haplochromis burtoni* | Burtoni | V1AR | Vasotocin | AAM70493^b^ |
| *Tetraodon nigroviridis* | Tetraodon | V1AR | Vasotocin | ENSTNIP00000009511^a^ |
| *Catostomus commersonii* | White sucker | V1AR | Vasotocin | Q90352^d^ |
| *Protopterus annectens* | West african lungfish | V1AR | Vasotocin | BAG66063^b^ |
| *Cynops pyrrhogaster* | Japanese fire belly newt | V1AR | Vasotocin | BAF38754^b^ |
| *Taricha granulosa* | Rough-skinned newt | V1AR | Vasotocin | ADF30857^b^ |
| *Takifugu rubripes* | Fugu | V1AR | Vasotocin | ENSTRUP00000014647^a^ |
| *Equus caballus* | Horse | V1BR | Vasopressin 1B | ENSECAP00000011404^a^ |
| *Pan troglodytes* | Chimpanzee | V1BR | Vasopressin 1B | ENSPTRP00000042684^a^ |
| *Loxodonta africana* | Elephant | V1BR | Vasopressin 1B | ENSLAFP00000010133^a^ |
| *Mus musculus* | Mouse | V1BR | Vasopressin 1B | ENSMUSP00000027690^a^ |
| *Cavia porcellus* | Guinea Pig | V1BR | Vasopressin 1B | ENSCPOP00000006139^a^ |
| *Gallus gallus* | Chicken | V1BR | Vasopressin 1B | NP_001026669^b^ |
| *Anolis carolinensis* | Anole lizard | V1BR | Vasopressin 1B | XP_003224056^b^ |
| *Monodelphis domestica* | Opossum | OTR-like | Oxytocin | XP_001375059^b^ |
| *Homo sapiens* | Human | OTR-like | Oxytocin | ENSP00000324270^a^ |
| *Tursiops truncatus* | Dolphin | OTR-like | Oxytocin | ENSTTRP00000005708^a^ |
| *Mus musculus* | Mouse | OTR-like | Oxytocin | ENSMUSP00000051132^a^ |
| *Gallus gallus* | Chicken | OTR-like | Oxytocin | NP_001026740^b^ |
| *Taricha granulosa* | Rough-skinned newt | OTR-like | Oxytocin | ABA27136^b^ |
| *Hyla japonica* | Japanese tree frog | OTR-like | Mesotocin | BAC23056^b^ |
| *Bufo marinus* | Marine toad | OTR-like |  | Q90252^d^ |
| *Rana catesbeiana* | Bullfrog | OTR-like | Mesotocin | AAQ22365^b^ |
| *Anolis carolinensis* | Anole lizard | OTR-like | Mesotocin | XP_003224939^b^ |
| *Catostomus commersonii* | White sucker | OTR-like | Isotocin | Q90334^d^ |
| *Oryzias latipes* | Medaka | OTR-like | Isotocin | ENSORLP00000004098^a^ |
| *Gasterosteus aculeatus* | Stickleback | OTR-like | Isotocin | ENSGACP00000001185^a^ |
| *Takifugu rubripes* | Fugu | OTR-like | Isotocin | ENSTRUP00000007547^a^ |
| *Tursiops truncatus* | Dolphin | V2R | Vasopressin 2 | ENSTTRP00000007052^a^ |
| *Mus musculus* | Mouse | V2R | Vasopressin 2 | ENSMUSP00000033765^a^ |
| *Homo sapiens* | Human | V2R | Vasopressin 2 | ENSP00000351805^a^ |
| *Anolis carolinensis* | Anole lizard | V2R | Vasopressin 2 | ENSACAP00000017745^a^ |
| *Gallus gallus* | Chicken | V2R | Vasopressin 2 | ENSGALP00000015444^a^ |
| *Cynops pyrrhogaster* | Japanese fire belly newt | V2R | Vasotocin 2 | BAF38755^b^ |
| *Taricha granulosa* | Rough-skinned newt | V2R | Vasotocin 2 | ABQ23253^b^ |
| ***Xenopus tropicalis*** | Western clawed frog | V2R | Vasotocin 2 | XP_002937279^b^ |
| *Bufo marinus* | Marine toad | V2R | Vasotocin 2 | BAF48113^b^ |
| *Hyla japonica* | Japanese tree frog | V2R | Vasotocin 2 | BAC2305 ^b^ |
| *Tribolium castaneum* | Red flour beetle | VPR | Inotocin | NP_001078830^b^ |
| *Acromyrmex echinatior* | Panamanian leafcutter ant | VPR | Vasotocin | EGI60623^b^ |
| *Eisenia fetida* | Common brandling worm | VPR | Annetocin | Q75W84^d^ |
| *Octopus vulgaris* | Octopus | VPR | Cephalotocin 1 | Q7YW31^d^ |
| *Octopus vulgaris* | Octopus | VPR | Cephalotocin 2 | Q5WA50^d^ |
| *Lymnaea stagnalis* | Great pond snail | VPR | Conopressin | AAA91998^b^ |
| *Saccoglossus kowalevskii* | Acorn worm | acornworm VPR | Vasotocin | XP_002735546^b^ |
| *Strongylocentrotus purpuratus* | Purple sea urchin | Seaurchin VPR | Vasopressin 1A | XP_784373^b^ |
| *Culex quinquefasciatus* | Southern house mosquito | mosquito CCAPR | Cardioacceleratory peptide | XP_001847670^b^ |
| *Drosophila melanogaster* | Fruit fly | fruit fly CCAPR | Cardioacceleratory peptide | AAN10041^b^ |
| *Tribolium castaneum* | Red flour beetle | rf beetle CCAPR | Cardioacceleratory peptide | NP_001076795^b^ |
| *Apis mellifera* | Honey bee | bee CCAPR^‡^ | Cardioacceleratory peptide | XP_001122652^b^ |
| *Daphnia Pulex* | Water flea | water flea CCAPR^+^ | Cardioacceleratory peptide | 317355^c^ |
| *Pediculus humanus corporis* | Human body louse | louse CCAPR^‡^ | Cardioacceleratory peptide | XP_002424871^b^ |
| *Capitella teleta* | Marine worm | marine worm CCAPR*^+^ | Cardioacceleratory peptide | 136071^c^ |
| *Lottia gigantea* | Owl limpet | limpet CCAPR*^+^ | Cardioacceleratory peptide | 126859^c^ |
| *Homo sapiens* | Human | human NPSR | Neuropeptide S | ENSP00000352839^a^ |
| *Gallus gallus* | Chicken | chicken NPSR | Neuropeptide S | ENSGALP00000019838^a^ |
| *Tursiops truncatus* | Dolphin | dolphin NPSR* | Neuropeptide S | ENSTTRP00000015651^a^ |
| *Pteropus vampyrus* | Megabat | megabat NPSR | Neuropeptide S | ENSPVAP00000016321^a^ |
| *Mus musculus* | Mouse | mouse NPSR | Neuropeptide S | ENSMUSP00000056432^a^ |
| *Monodelphis domestica* | Opossum | opossum NPSR | Neuropeptide S | XP_001365641^b^ |
| *Anolis carolinensis* | Anole lizard | lizard NPSR* | Neuropeptide S | XP_003222313^b^ |
| ***Xenopus tropicalis*** | Western clawed frog | frog NPSR | Neuropeptide S | XP_002937035^b^ |
| *Branchiostoma floridae* | Florida lancelet | lancelet NPSR-like*^+^ | Putative NG peptide | 212588^c^ |
| *Saccoglossus kowalevskii* | Acorn worm | acorn worm NPSR-like^+^ | Putative NG peptide | XP_002732566^b^ |
| *Branchiostoma floridae* | Florida lancelet | GnRHR1 | Gonadotropin releasing hormone receptor 1 | ACC68665^b^ |
| *Branchiostoma floridae* | Florida lancelet | GnRHR2 | Gonadotropin releasing hormone receptor 2 | ACC68666^b^ |
| *Anolis carolinensis* | Anole lizard | GnRHR IIa | Gonadotropin releasing hormone receptor | ENSACAP00000004352^a^ |
| *Eublepharis macularius* | Leopard gecko | GnRHR IIa | Gonadotropin releasing hormone receptor | BAD11150^b^ |
| *Rana catesbeiana* | Bullfrog | GnRHR IIa | Gonadotropin releasing hormone receptor 2 | AAG42949^b^ |
| *Xenopus tropicalis* | Western clawed frog | GnRHR IIa | Gonadotropin releasing hormone receptor 2 | NP_001107548^b^ |
| *Clarias gariepinus* | North african catfish | GnRHR IIa | Gonadotropin releasing hormone receptor 1 | CAA66128^b^ |
| *Danio rerio* | Zebrafish | GnRHR IIa | Gonadotropin releasing hormone receptor 1 | NP_001138452^b^ |
| *Clarias gariepinus* | North african catfish | GnRHR IIa | Gonadotropin releasing hormone receptor 2 | AAM95605^b^ |
| *Oryzias latipes* | Medaka | GnRHR IIa | Gonadotropin releasing hormone receptor 2 | NP_001098392^b^ |
| *Odontesthes bonariensis* | Pejerrey | GnRHR IIa | Gonadotropin releasing hormone receptor 1 | ABI75337^b^ |
| *Danio rerio* | Zebrafish | GnRHR IIa | Gonadotropin releasing hormone receptor 3 | NP_001170921^b^ |
| *Rana catesbeiana* | Bullfrog | GnRHR IIb | Gonadotropin releasing hormone receptor 1 | AAG42575^b^ |
| *Xenopus tropicalis* | Western clawed frog | GnRHR IIb | Gonadotropin releasing hormone receptor 3 | NP_001107548^b^ |
| *Oryzias latipes* | Medaka | GnRHR IIb | Gonadotropin releasing hormone receptor 1 | NP_001098352^b^ |
| *Oryzias latipes* | Medaka | GnRHR IIb | Gonadotropin releasing hormone receptor 3 | NP_001098393^b^ |
| *Danio rerio* | Zebrafish | GnRHR IIb | Gonadotropin releasing hormone receptor 4 | NP_001091663^b^ |
| *Odontesthes bonariensis* | Pejerrey | GnRHR IIb | Gonadotropin releasing hormone receptor 2 | ABI75336^b^ |
| *Danio rerio* | Zebrafish | GnRHR IIb | Gonadotropin releasing hormone receptor 2 | NP_001138451^b^ |
| *Rana catesbeiana* | Bullfrog | GnRHR IIb | Gonadotropin releasing hormone receptor 3 | AAG42574^b^ |
| *Xenopus laevis* | African clawed frog | GnRHR IIb | Gonadotropin releasing hormone receptor 3 | NP_001107549.1^b^ |
| *Taeniopygia guttata* | Zebra finch | GnRHR IIb | Gonadotropin releasing hormone receptor | XP_002190060^b^ |
| *Macaca mulatta* | Macaque | GnRHR IIb | Gonadotropin releasing hormone receptor 2 | NP_001028014^b^ |
| *Callithrix jacchus* | Marmoset | GnRHR IIb | Gonadotropin releasing hormone receptor 2 | XP_002759859^b^ |
| *Sus scrofa* | Pig | GnRHR IIb | Gonadotropin releasing hormone receptor 2 | NP_001001639^b^ |
| *Tupaia belangeri* | Tree shrew | GnRHR IIb | Gonadotropin releasing hormone receptor 2 | ENSTBEP00000013008^a^ |
| *Monodelphis domestica* | Opossum | GnRHR IIb | Gonadotropin releasing hormone receptor 2 | XP_001369168^b^ |
| *Bos taurus* | Cow | GnRHR 1 | Gonadotropin releasing hormone receptor | NP_803480^b^ |
| *Tursiops truncatus* | Dolphin | GnRHR 1 | Gonadotropin releasing hormone receptor | ENSTTRP00000007374^a^ |
| *Homo sapiens* | Human | GnRHR 1 | Gonadotropin releasing hormone receptor | NP_000397^b^ |
| *Macaca mulatta* | Macaque | GnRHR 1 | Gonadotropin releasing hormone receptor 1 | XP_001109227^b^ |
| *Callithrix jacchus* | Marmoset | GnRHR 1 | Gonadotropin releasing hormone receptor 1 | XP_002745835^b^ |
| *Sus scrofa* | Pig | GnRHR 1 | Gonadotropin releasing hormone receptor 1 | NP_999438^b^ |
| *Mus musculus* | Mouse | GnRHR 1 | Gonadotropin releasing hormone receptor | NP_034453^b^ |
| *Tupaia belangeri* | Tree shrew | GnRHR 1 | Gonadotropin releasing hormone receptor 1 | ENSTBEP00000014843^a^ |
| *Cavia porcellus* | Guinea pig | GnRHR 1 | Gonadotropin releasing hormone receptor | NP_001166428^b^ |
| *Monodelphis domestica* | Opossum | GnRHR1 | Gonadotropin releasing hormone receptor 1 | XP_001362289^b^ |
| *Ornithorhynchus anatinus* | Platypus | GnRHR1 | Gonadotropin releasing hormone receptor | NP_001116830^b^ |
| *Tribolium castaneum* | Red flour beetle | ACPR | Adipokinetic hormone / corazonin –related receptor | ABX52400^b^ |
| *Nasonia vitripennis* | Jewel wasp | ACPR | Adipokinetic hormone / corazonin –related receptor | NP_001164571^b^ |
| *Anopheles gambiae* | Mosquito | ACPR | Adipokinetic hormone / corazonin –related receptor | ABX52399^b^ |
| *Bombyx mori* | Silkworm | ACPR | Adipokinetic hormone / corazonin –related receptor | NP_001127745^b^ |
| *Apis mellifera* | Honey bee | AKHR | Adipokinetic Hormone receptor | NP_001035354^b^ |
| *Tribolium castaneum* | Red flour beetle | AKHR | Adipokinetic Hormone receptor | NP_001076809^b^ |
| *Nasonia vitripennis* | Jewel wasp | AKHR | Adipokinetic Hormone receptor | NP_001161243^b^ |
| *Anopheles gambiae* | Mosquito | AKHR | Adipokinetic Hormone receptor | XP_308034^b^ |
| *Manduca sexta* | Moth | AKHR | Adipokinetic Hormone receptor | ACE00761^b^ |
| *Periplaneta americana* | Cockroach | AKHR | Adipokinetic Hormone receptor | ABB20590^b^ |
| *Bombyx mori* | Silkworm | AKHR | Adipokinetic Hormone receptor | NP_001037049^b^ |
| *Drosophila melanogaster* | Fruit fly | AKHR | Adipokinetic Hormone receptor | NP_477387^b^ |
| *Capitella teleta* | Marine worm | AKHR | Adipokinetic Hormone receptor | 125249^c^ |
| *Crassostrea gigas* | Oyster | AKHR | Adipokinetic Hormone receptor | CAI64587^b^ |
| *Caenorhabditis briggsae* | Cbriggsae | AKHR | Adipokinetic Hormone receptor1 | XP_002639368^b^ |
| *Caenorhabditis briggsae* | Cbriggsae | AKHR | Adipokinetic Hormone receptor2 | XP_002637834^b^ |
| *Caenorhabditis elegans* | Celegans | AKHR | Adipokinetic Hormone receptor1 | NP_491453^b^ |
| *Caenorhabditis elegans* | Celegans | AKHR | Adipokinetic Hormone receptor2 | NP_506566^b^ |
| *Caenorhabditis elegans* | Celegans | AKHR | Adipokinetic Hormone receptor3 | NP_509685^b^ |
| *Caenorhabditis remanei* | Cremanei | AKHR | Adipokinetic Hormone receptor3 | XP_003102575^b^ |
| *Branchiostoma floridae* | Florida lancelet | GnRHR3 | Gonadotropin releasing hormone receptor 3 | ACC68668^b^ |
| *Branchiostoma floridae* | Florida lancelet | GnRHR4 | Gonadotropin releasing hormone receptor 4 | ACN79527^b^ |
| *Octopus vulgaris* | Octopus | GnRHR | Gonadotropin releasing hormone receptor | Q2V2K5^d^ |
| *Apis mellifera* | Honey bee | CrzR | Corazonin receptor | NP_001137393^b^ |
| *Daphnia pulex* | Water flea | CrzR | Corazonin receptor | 312207^c^ |
| *Drosophila melanogaster* | Fruit fly | CrzR | Corazonin receptor | NP_648571^b^ |
| *Manduca sexta* | Moth | CrzR | Corazonin receptor | AAR14318^b^ |
| *Bombyx mori* | Silkworm | CrzR | Corazonin receptor | NP_001127719^b^ |
| *Ciona intestinalis* | Tunicate | GnRHR | Gonadotropin releasing hormone receptor 1 | NP_001028997^b^ |
| *Ciona intestinalis* | Tunicate | GnRHR | Gonadotropin releasing hormone receptor 2 | NP_001028996^b^ |
| *Ciona intestinalis* | Tunicate | GnRHR | Gonadotropin releasing hormone receptor 3 | NP_001028995^b^ |
| *Homo sapiens* | Human | GALR | Galanin receptor type 1 | P47211^d^ |
| *Homo sapiens* | Human | GALR | Galanin receptor type 2 | O43603^d^ |
| *Rattus norvegicus* | Rat | GALR | Galanin receptor type 2 | O08726^d^ |
| *Mus musculus* | Mouse | GALR | Galanin receptor type 3 | O88853^d^ |
| *Mus musculus* | Mouse | KISSR | KiSS-1 receptor | Q91V45^d^ |
| *Homo sapiens* | Human | KISSR | KiSS-1 receptor | Q969F8^d^ |
| *Danio rerio* | Zebrafish | SSTR | Somatostatin receptor type 5 | ENSDARP00000103540^a^ |
| *Homo sapiens* | Human | SSTR | Somatostatin receptor type 1 | P30872^d^ |
| *Gasterosteus aculeatus* | Stickleback | SSTR | Somatostatin receptor type 1 | ENSGACP00000000999^a^ |
| *Mus musculus* | Mouse | SSTR | Somatostatin receptor type 4 | P49660^d^ |
| *Homo sapiens* | Human | SSTR | Somatostatin receptor type 4 | P31391^d^ |
| ***Otolemur garnettii*** | Bush baby | SSTR | Somatostatin receptor type 4 | ENSOGAP00000015318^a^ |
| *Gasterosteus aculeatus* | Stickleback | SSTR | Somatostatin receptor type 4 | ENSGALP00000013602^a^ |
| *Homo sapiens* | Human | SSTR | Somatostatin receptor type 2 | P30874^d^ |
| *Xenopus laevis* | African clawed frog | SSTR | Somatostatin receptor type 2 | Q6GQ36^d^ |
| *Gallus gallus* | Chicken | SSTR | Somatostatin receptor type 2 | Q58G84^d^ |
| *Rattus norvegicus* | Rat | SSTR | Somatostatin receptor type 5 | P30938^d^ |
| *Mus musculus* | Mouse | SSTR | Somatostatin receptor type 3 | P30935^d^ |
| *Homo sapiens* | Human | SSTR | Somatostatin receptor type 3 | P32745^d^ |
| *Canis lupus familiaris* | Dog | SSTR | Somatostatin receptor type 3 | Q4L144^d^ |
| ***Xenopus tropicalis*** | Western clawed frog | SSTR | Somatostatin receptor type 3 | ENSXETP00000034975^a^ |
| *Takifugu rubripes* | Fugu | SSTR | Somatostatin receptor type 3 | ENSTRUP00000019241^a^ |
| *Tetraodon nigroviridis* | Tetraodon | SSTR | Somatostatin receptor type 4 | ENSTNIP00000006082^a^ |
| *Takifugu rubripes* | Fugu | SSTR | Somatostatin receptor type 5 | O42179^d^ |
| *Mus musculus* | Mouse | TACR | Neuromedin-K receptor3 | P47937 ^d^ |
| *Oryctolagus cuniculus* | Rabbit | TACR | Neuromedin-K receptor3 | **O97512**^d^ |
| *Cavia porcellus* | Guinea Pig | TACR | Neuromedin-K receptor3 | P30098^d^ |
| *Tetraodon nigroviridis* | Tetraodon | TACR | Neuromedin-K receptor3 | Q4SDK8^d^ |
| *Cavia porcellus* | Guinea Pig | TACR | Neuromedin-K receptor1 | P30547^d^ |
| *Rana catesbeiana* | Bullfrog | TACR | Neuromedin-K receptor1 | **Q98982**^d^ |
| *Mus musculus* | Mouse | TACR | Neuromedin-K receptor2 | **P30549**^d^ |
| *Homo sapiens* | Human | TACR | Neuromedin-K receptor2 | **P21452**^d^ |
| *Bos taurus* | Cow | TACR | Neuromedin-K receptor2 | **P05363**^d^ |
| *Cavia porcellus* | Guinea Pig | TACR | Neuromedin-K receptor2 | **Q64077**^d^ |
| *Canis lupus familiaris* | Dog | TACR | Neuromedin-K receptor2 | Q5DUB2^d^ |
| *Anopheles gambiae* | Mosquito | TACR | Tachykinin receptor2 | Q8T5J7^d^ |
| *Anopheles gambiae* | Mosquito | TACR | Tachykinin receptor1 | **Q7PRC5**^d^ |
| *Drosophila willistoni* | Drosophila | TACR | Tachykinin receptor1 | B4NAZ4^d^ |
| *Gallus gallus* | Chicken | NPFFR | Neuropeptide FF type receptor 1 | Q75XU5^d^ |
| *Taeniopygia guttata* | Zebra finch | NPFFR | Neuropeptide FF type receptor 1 | XP_002192220^b^ |
| ***Oryzias latipes*** | Medaka | NPFFR | Neuropeptide FF type receptor 1 | ENSORLP00000690069^a^ |
| *Rattus norvegicus* | Rat | NPFFR | Neuropeptide FF type receptor 1 | Q9EP86^d^ |
| *Bos taurus* | Cow | NPFFR | Neuropeptide FF type receptor 1 | ENSBTAP00000011957^a^ |
| ***Loxodonta africana*** | Elephant | NPFFR | Neuropeptide FF type receptor 1 | ENSLAFP00000003379^a^ |
| *Gallus gallus* | Chicken | NPFFR | Neuropeptide FF type receptor2 | NP_001029997^b^ |
| *Xenopus tropicalis* | Western clawed frog | NPFFR | Neuropeptide FF type receptor 1 | XP_002936011^b^ |
| ***Gasterosteus aculeatus*** | Stickleback | NPFFR | Neuropeptide FF receptor type 1 | ENSGACP00000019104^a^ |
| ***Tetraodon nigroviridis*** | **Tetraodon** | NPFFR | Neuropeptide FF receptor type 1 | ENSTNIP00000018411^a^ |
| ***Oryzias latipes*** | Medaka | NPFFR | Neuropeptide FF receptor type 2 | ENSORLP00000003808^a^ |
| ***Takifugu rubripes*** | Fugu | NPFFR | Neuropeptide FF receptor type 2 | ENSTRUP00000047242^a^ |
| ***Tetraodon nigroviridis*** | **Tetraodon** | NPFFR | Neuropeptide FF receptor type 2 | ENSTNIP00000010296^a^ |
| ***Gasterosteus aculeatus*** | Stickleback | NPFFR | Neuropeptide FF receptor type 2 | ENSGACP00000020936^a^ |
| *Mus musculus* | Mouse | NPFFR | Neuropeptide FF receptor type 2 | Q924N0^d^ |
| *Taeniopygia guttata* | Zebra finch | NPFFR | Neuropeptide FF type receptor 2 | XP_002187367^b^ |
| *Xenopus tropicalis* | Western clawed frog | NPFFR | Neuropeptide FF type receptor 2 | ENSXETP00000017794^a^ |
| *Monodelphis domestica* | Opossum | NPFFR | Neuropeptide Y | ENSMODP00000023858^a^ |
| *Xenopus tropicalis* | Western clawed frog | NPFFR | Neuropeptide FF type receptor 2 | NP_001107549 ^b^ |
| *Taeniopygia guttata* | Zebra finch | NPFFR | Neuropeptide FF type receptor 2 | XP_002193325 ^b^ |
| *Branchiostoma floridae* | Florida lancelet | NPFFR | Neuropeptide FF type receptor 2 | XP_002610318 ^b^ |
| *Caenorhabditis briggsae* | Cbriggsae | NPFFR 1 | Neuropeptide FF type receptor 1 | XP_002646062^b^ |
| *Caenorhabditis briggsae* | Cbriggsae | NPFFR 2 | Neuropeptide FF type receptor 2 | A8WSK3^d^ |
| *Caenorhabditis briggsae* | Cbriggsae | NPFFR | Neuropeptide FF type receptor 2 | XP_002643383^b^ |
| *Caenorhabditis briggsae* | Cbriggsae | NPFFR | Neuropeptide FF type receptor 2 | XP_002641465^b^ |
| *Caenorhabditis briggsae* | Cbriggsae | NPFFR | Neuropeptide FF type receptor 2 | XP_002643427^b^ |
| *Caenorhabditis elegans* | Celegans | NPFFR 2 | Neuropeptide FF type receptor 2 | O02043^d^ |
| *Caenorhabditis elegans* | Celegans | NPFFR | Neuropeptide FF type receptor 2 | NP_497744^b^ |
| *Caenorhabditis elegans* | Celegans | NPFFR | Neuropeptide FF type receptor 2 | Q03613^d^ |
| *Mus musculus* | Mouse | NPYR | Neuropeptide Y receptor type 6 | Q61212^d^ |
| *Oryctolagus cuniculus* | Rabbit | NPYR | Neuropeptide Y receptor type 6 | P79217^d^ |
| *Gallus gallus* | Chicken | NPYR | Neuropeptide Y | NP_001038152 ^b^ |
| *Oryzias latipes* | Medaka | NPYR | [Neuropeptide Y / peptide YY](http://www.gpcr.org/7tm/proteinfamily/001_002_014_006) | ENSORLP00000011957^a^ |
| *Homo sapiens* | Human | NPYR | Neuropeptide Y receptor type 1 | P25929^d^ |
| *Meleagris gallopavo* | Turkey | NPYR | Neuropeptide Y receptor Y1 | XP_003205420 ^b^ |
| *Danio rerio* | Zebra fish | NPYR | Neuropeptide Y receptor Y1 | ENSDARP00000054443^a^ |
| *Ornithorhynchus anatinus* | Platypus | NPYR | [Neuropeptide Y / peptide YY](http://www.gpcr.org/7tm/proteinfamily/001_002_014_006) | ENSOANP00000024118^a^ |
| *Mus musculus* | Mouse | NPYR | Neuropeptide Y receptor type 4 | Q8BZF9^d^ |
| *Homo sapiens* | Human | NPYR | Neuropeptide Y receptor type 4 | XP_001129329 ^b^ |
| *Gallus gallus* | Chicken | NPYR | Neuropeptide Y receptor type 4 | Q8QGM3^d^ |
| *Bos taurus* | Cow | NPYR | Neuropeptide Y receptor type 4 | ENSBTAP00000006247^a^ |
| *Oryctolagus cuniculus* | Rabbit | NPYR | Neuropeptide Y receptor type 4 | Q2RGM3^d^ |
| *Gallus gallus* | Chicken | NPYR | Neuropeptide Y receptor type 4 | NP_001026726 ^b^ |
| *Takifugu rubripes* | Fugu | NPYR | Neuropeptide Y | NP_001098075 ^b^ |
| *Mus musculus* | Mouse | NPYR | Neuropeptide Y receptor type 2 | Q3SWR9^d^ |
| *Gallus gallus* | Chicken | NPYR | Neuropeptide Y receptor type 2 | **Q9DDN6**^d^ |
| ***Xenopus tropicalis*** | Western clawed frog | NPYR | Neuropeptide Y receptor type 2 | ENSXETP00000058101^a^ |
| *Gasterosteus aculeatus* | Stickleback | NPYR | Neuropeptide Y receptor type 2 | ENSGACP00000024225^a^ |
| *Tetraodon nigroviridis* | Tetraodon | NPYR | Neuropeptide Y receptor type 2 | ENSTNIP00000000731^a^ |
| *Mus musculus* | Mouse | NPYR | Neuropeptide Y receptor type 5 | ENSMUSP00000065157 ^a^ |
| *Canis lupus familiaris* | Dog | NPYR | Neuropeptide Y receptor type 5 | O62729^d^ |
| *Gallus gallus* | Chicken | NPYR | Neuropeptide Y receptor Y5 | Q8QFM2^d^ |
| *Culex quinquefasciatus* | Culex mosquito | NPYR | Neuropeptide Y receptor type | B0wj64^d^ |
| *Drosophila melanogaster* | Fruit Fly | NPYR | Neuropeptide Y receptor type 1 | Q9VNM1^d^ |
| *Mus musculus* | Mouse | NMUR | Neuromedin-U receptor 2 | Q8BZ39^d^ |
| *Homo sapiens* | Human | NMUR | Neuromedin-U receptor 2 | Q9GZQ4^d^ |
| *Bos taurus* | Cow | NMUR | Neuromedin-U receptor 2 | Q58CW4^d^ |
| *Gallus gallus* | Chicken | NMUR | Neuromedin-U receptor 2 | E1BX29^d^ |
| *Taeniopygia guttata* | Zebra finch | NMUR | Neuromedin-U receptor 2 | XP_002195061^b^ |
| *Gallus gallus* | Chicken | NMUR | Neuromedin-U receptor 1 | XP_426705^b^ |
| *Taeniopygia guttata* | Zebra finch | NMUR | Neuromedin-U receptor 1 | XP_002190989^b^ |
| *Danio rerio* | Zebrafish | NMUR | Neuromedin-U receptor 1 | XP_698473^b^ |
| *Mus musculus* | Mouse | NMUR | Neuromedin-U receptor 1 | O55040^d^ |
| *Rattus norvegicus* | Rat | NMUR | Neuromedin-U receptor 1 | Q9JJI5^d^ |
| *Equus caballus* | Horse | NMUR | Neuromedin-U receptor 1 | XP_001495230^b^ |
| *Pan troglodytes* | Chimpanzee | NMUR | Neuromedin-U receptor 1 | XP_00114047^b^ |
| *Monodelphis domestica* | Opossum | NMUR | Neuromedin-U receptor 1 | XP_001373154^b^ |
| *Gasterosteus aculeatus* | Stickleback | NTSR | Neurotensin receptor type1 | ENSGACP00000005851^a^ |
| *Mus musculus* | Mouse | NTSR | Neurotensin receptor type1 | O88319^d^ |
| *Homo sapiens* | Human | NTSR | Neurotensin receptor type1 | P30989^d^ |
| *Loxodonta africana* | Elephant | NTSR | Neurotensin receptor type1 | ENSLAFP00000007444^a^ |
| *Homo sapiens* | Human | NTSR | Neurotensin receptor type2 | O95665^d^ |
| ***Felis catus*** | Cat | NTSR | Neurotensin receptor type 2 | ENSFCAP00000007897^a^ |
| *Tupaia belangeri* | Tree shrew | NTSR | Neurotensin receptor type 2 | ENSTBEP00000014579^a^ |
| *Rattus norvegicus* | Rat | NTSR | Neurotensin receptor type 2 | Q63384^d^ |

Note: The common and binomial names of the species, the names, abbreviations and accession numbers of the sequences are listed.

^a^ Sequence from Ensembl database,  ^b^ Sequence from NCBI database, ^c^ Sequence from JGI database and ^d^ Sequence from UniProt–Protein Knowledgebase.

Sequences corrected manually at N and C- termini are represented with * and ^+^ for NPSR, NPSR-like and CCAPR at the end of the name in the abbreviation used for phylogeny column.

Fragmented CCAPR sequences are represented with ^‡^ at the end of the name in the abbreviation used for phylogeny column.
