# Supplementary material for: Molecular Evolution of the Neuropeptide S Receptor
Source: PLoS One. 2012 Mar 30;7(3):e34046. doi: 10.1371/journal.pone.0034046 (PMC3316597; doi:10.1371/journal.pone.0034046)
Supplement: Data S1 — Amino acid sequences of NPSR and NPSR-like sequences in FASTA format. (DOC) [file pone.0034046.s007.doc]

**Data S1.** Amino acid sequences of NPSR and NPSR-like sequences in FASTA format

>lizard NPSR*

MDSTFIYIISESAYKHHSHKQCPVSLFGHKCPCFLYVVTLYSTYGRKVTYIDHDFV

QTEQLVTLWILFVLTVVGNSIVLLSIWKRKRKSRMTFFVTQLAITDCFTGLINIMTDIIW

RFTGDFMAPDLVCRIVRYFQVVLLYASTYVLVSLSIDRYHAIVYPMKFFQGERQAKILIV

IAWGLSFLFSIPTLIIFGKRQLSNGEVQCWAVWPDDSYWIPYMTTVAFLVYFIPLIIISV

IYFIVIRTIWTKSKAHAIIVSNCSDGKFCTSYTHRGLISKAKIKAVKYSAVIILAFVLCW

SPYFLFDILDNFSILPETKERFYAAVIIQNLPALNSAINPIIYCIFSDTLCQAFRSRRRS

GNLGTFRERTEGQEMQVLSKPEYI

>bush baby NPSR

MPANLTEGSPNSGGTTQTPAPSLVACTDTVTFTEVAEGEEWGSFYYSFKTEQLITLWVLF

VFTIVGNSIVLFSTCRRKRKSRMTFFVTQLAITDSFTGLVNILTDIIWRFTGDFMAPDLV

CRVVRYLQVVLLYASTYVLVSLSIDRYHAIVYPMKFPQGVKQAKVLIIIAWILSFLFSIP

TLIIFGKRTLSNGEVQCWALWPDDSYWTPYMTIVAFLVYFIPLTIISVIYGIVIRTIWIK

SKAHDSVISNCSGGKLCTSYNRGLISKAKIKAIKYSIVIILAFICCWSPYFLFDILDNFS

FFPDTKERFYASVIIQNLPALRRINPLVYCAFSSICPWGANSSAYLLADDASVLTWVLTS

GKEGCERKEAKITGFQNDIPRENR

>marmoset NPSR

MPANFTESSLDSNGTRQTPDSSPVACTETVTFTEVVEGKEWGSFYYSFKTEQLITLWVLF

VFTIVGNSVVLFSTWRRKRKSRMIFFVTQLAITDSFTGLVNILTDIIWRFTGDFVAPDLV

CRVVRYLQVVLLYASTYVLVSLSIDRYHAIVYPMKFLQGEKQARVLIVIAWGLSFLFSIP

TLIIFGKRTLSNGEVQCWALWPDDSYWTPYMTVVAFLVYFIPLTIISVMYGIVIRTIWIK

SKTYESVISNCSDGKLCSRYNRGPISKAKLKAIKYSIVIILAFICCWSPYFLFDILDNFN

LLPDTQERFYASVIIQNLPALNSAMNPLIYCVFSSSISFPCRASSTVYLLACDVAVLWGL

VLPGGKENCESLRRKGAKITGFQNDVPGENR

>chicken NPSR

MEVGPTAASSSSPAGTGWLDVTLVTCTDTVTFTEVLEEEEWKSFYYSFKTEQLVTLWILF

IVTIAGNAIVLFSTWRRKRKSRMTFFVTQLAITDSFTGLINIMTDIIWRYTGDFMAPDIV

CRVVRYLQVVLLYASTYVLVSLSIDRYHAIVYPMKFMQGERQAKVLIGVAWSLSFLFSIP

TLIIFGKRQLSNGEVQCWALWPDDSYWIPYMTVVAFLVYFIPLIIISVIYSIVIRTIWMK

SKAQAVIVSSCTDGKTSAGYTSRGFISRAKVKAIKYSIVIVLAFALCWSPYFLFDILDNF

NILPETKERFYASVIIQNLPALNSAINPLIYCLFSNHLCTPFEERRTRRLEGTFRDRSDG

GQEMQVLSKPEYI

>chimpanzee NPSR

MPANFTEGSFDSSGTGQTLDSSPVACTETVTFTEVVEGKEWGSFYYSFKTEQLITLWVLF

VFTIVGNSVVLFSTWRRKKKSRMTFFVTQLAITDSFTGLVNILTDIIWRFTGDFMAPDLV

CRVVRYLQVVLLYASTYVLVSLSIDRYHAIVYPMKFLQGEKQARVLIVIAWSLSFLFSIP

TLIIFGKRTLSNGEVQCWALWPDDSYWTPYMTIVAFLVYFIPLTIISIMYGIVIRTIWIK

SKTYETVISNCSDGKLCSSYNRGLISKAKIKAIKYSIIIILAFVCCWSPYFLFDILDNFN

LLPDTQERFYASVIIQNLPALNSAINPLIYCVFSSSISFPCRVIPLRQLQEAALTLCPQR

QNWKGTWPGVPSWALPR

>dolphin NPSR*

MPANLTEGSPKSIGTGQTLDSPILCTETVTFTAVVEGEERGSFYYSFKTEQLITLWVLCVFTIVG

NAIMLFSTLRRKRKSRMTFVAQLAITDSFTGLVNILTDIIGRFTGDFMAPDLVCVVHYVQ

VVLLYASTYVLGSLRIDRYHAIIYPMKFLQGEKQAKVLSVIAWSFSFLFSIPTLIIFGKR

KLSNGEVQCWALPDDSYQTPHMTIVAFLVYFIPLTIISVIYAIVIRMIWVKSKAHETEIS

SCSDGKRCTISNGLISKAKINAIKYSIVIILAFICCWSPYFLFDILDNFSLLPNTKERFY

ASVIIQNLPALNNAINPLIYCVFSSSICFPCGANSSAYLLASDVSVHWALVLTSRKEGCE

WWGRK

>guinea Pig NPSR

MPSHRPQDHSSIVAQTAPSLDASSLDAALVACTDTVTFTEVVEGETWGSFYSSYKTEQLITLWVLFVFTISGNSVLLFSTWKRKRKSRMTFFVTQLAITDSFTGLVNILTDIIWRFTGDFLAPDLVCRVVRYLQVVLLYASTYVLVSLSIDRYHAIVYPMKFLQGERQAKVLIGVAWSLSFLFSIPTLIIFGKRTLSNGEVQCWALWPDDSYWTPYMTVVAVLVYFIPLTILSIIYSIVIRTIWMKSKAPETVISNYSDGKLCTSYNRGFISKAKVKAIKYSIVIVLAFTICWSPYFLFDILDNFNLLPDTKERFYASVIIQNLPALNSAINPLIYCIFNRPICFPCREQKSQVSRMTCRERSERHELQFLSKPEFI

>horse NPSR

MPANLTEGSPNSNGTMQTLDSSPVLCTGTVTFTEVVEGEEWGSFYYSFRTEQLITLWVLF

VFTIVGNSIVLFSIWRRKRKSRMTFFVTQLAITDSFTGLVNILTDIIWRFTGDFMAPDLV

CRVVRYLQVVLLYASTYVLVSLSIDRYHAIVYPMKFLQGEKQARVLIVIAWGLSFLFSIP

TLIIFGKRKLPSGEVQCWALWPDDSYWTPYMTIVAFLVYFIPLTIISVIYAIVIRTIWTK

SKASETVISNCSDGKLCTSYNRGLISKAKIKAIKYSIVIILAFICCWSPYFLFDILDNFS

LLPDTKERFYASVIIQNLPALNSAINPLIYCVFSSSICFPCGDQRSQDSRMTYQERTERH

EMQVLSKPEFI

>human NPSR

MPANFTEGSFDSSGTGQTLDSSPVACTETVTFTEVVEGKEWGSFYYSFKTEQLITLWVLF

VFTIVGNSVVLFSTWRRKKKSRMTFFVTQLAITDSFTGLVNILTDINWRFTGDFTAPDLV

CRVVRYLQVVLLYASTYVLVSLSIDRYHAIVYPMKFLQGEKQARVLIVIAWSLSFLFSIP

TLIIFGKRTLSNGEVQCWALWPDDSYWTPYMTIVAFLVYFIPLTIISIMYGIVIRTIWIK

SKTYETVISNCSDGKLCSSYNRGLISKAKIKAIKYSIIIILAFICCWSPYFLFDILDNFN

LLPDTQERFYASVIIQNLPALNSAINPLIYCVFSSSISFPCRVIRLRQLQEAALMLCPQR

ENWKGTWPGVPSWALPR

>macaque NPSR

MPANFTEGSFDSNGTGQMLDSSPVACTETVTFTEVVEGKEWGSFYYSFKTEQLITLWVLF

VFTIVGNSVVLFSTWRRKRKSRMTFFVTQLAITDSFTGLVNILTDIIWRFTGDFMAPDLV

CRVVRYLQVVLLYASTYVLVSLSIDRYHAIVYPMKFLQGEKQAKVLIVIAWSLSFLFSIP

TLIIFGKRTLSNGEVQCWALWPDDSYWTPYMTIVAFLVYFIPLTIISVMYGIVIRTIWIK

SKTYETVISNCSDGKLCSSYNRGLISKAKIKAIKYSIVIILAFICCWSPYFLFDILDNFN

LLPDTQERFYASVIIQNLPALNSAINPLIYCVFSSSISFPCGERRSQDSRMTFRERTERH

EMQILSKPEFI

>megabat NPSR

MPANLTEGSPNSNGTMQTLDSSTATCFPTTLQEVEEEEWGSFYYSLKTEQLITLWVLFVF

TIVGNAIVLFSTWRRKRKSRMTFFVTQLAVTDSFTGLVNILTDIIWRFTGDFLAPDLVCR

VVRYLQVVLLYASTYVLVSLSIDRYHAIVYPMNFLQGEKQAKILTVTAWSLSFLFSIPTL

IIFGKRKLSNGEVQCWALWPDDSYWTPYMTIVAFLVYFIPLAIISVIYAIVIQTIWMKSK

GHDESVISNCSDGKLCTSYNRGLISKAKIKAIKYSMVIILAFICCWSPYFLFDMLDNFGL

LPDTEERFYASVIIQNLPALNSAINPLIYCAFSSSLCFPRRVNSSAYLLASAVIHWALVL

GSGGKAKITGFQDDVPRENR

>mouse NPSR

MPANLTEGSFHANQTVPMLDSSPVACTEIVTFTEALVAEEWGSFYSSFKTEQLITLWVLF

VVTIVGNSVVLFSTCRRKRKSRMTFFVTQLAITDSFTGLINILTDIIWRFTGDFMAPDLV

CRVVRYLQVVLLYASTYVLVSLSIDRYHAIVYPMKFLQGEKQAKVLIGIAWSLSFLFSIP

TLIIFGKRTLSNGEVQCWALWPDDSYWTPYMTIVAFLVYFIPLAIISVIYGLVIRTIWMK

SKTHETVISNCSDGKLCCSYNRGLISKAKIKAIKYSIVIILAFICCWSPYFLFDILDNFN

VLPDTKERFYASVIIQNLPALNSAINPLIYCIFSSSICSPCKMQRSQDSRMTYRERSERH

EMQILSKPEFI

>mouse lemur NPSR

MAAHLTEGSPNSSGTTQTLDSSPVACTETVTVTEVVEGEEWGSFYYSFKTEQLITLWVLF

VFTIVGNSVVLFSTWRRKRKSRMTFFVTQLAITDTFTGLVNILTDIIWRFTGDFMAPDLV

CRVVRYLQVVLLYASTYVLVSLSIDRYHAIVYPMKFLQGEKQAKVLIMIAWILAFLFSIP

TLIIFGKRTLSNGEVQCWALWPDDSYIPYMTIVXFLVYFIPLTIISVIYGIVIRTIWIKS

KAHETVISNCSGGKLCTSYNRGLISKAKIKAIKYSIVIILAFICCWSPYFLFDILDNFNL

LPDTKERFYASVIIQNLPALNSAINPLIYCAFSSSICFPWGTRNSVYLLACDASSVHWAV

LTSGKEGCEWRKKGAKITGFQSDIPGEDG

>rat NPSR

MPANLTEGSFHANQTVPMLDSSPVACTEIVTFTEALEAEEWGSFYSSFKTEQLITLWVLF

VFTIVGNSVVLFSTWRRKRKSRMTFFVTQLAITDSFTGLINILTDIIWRFTGDFMAPDLV

CRIVRYLQVVLLYASTYVLVSLSIDRYHAIVYPMKFLQGAEKQAKVLIGIAWSLSFLFSI

PTLIIFGKRTLSNGEVQCWALWPDDSYWTPYMTIVAFLVYFIPLTIISVIYGLVIRTIWI

KSKAHETVISNCSDGELCCSYNRGLISKAKIKAIKYSIVIILAFICCWSPYFLFDMLDNF

NLLPDTKERFYASVIIQNLPALNSAINPLIYCIFSGSLCSPCKVQRSQDSRMTYRERSER

HEMQILSKPEFI

>zebra finch NPSR

MEVNSTEASSSSQGGTSCLDLTLVTFTETVTFTEVVAEEEWGSFYYSFKTEQLVTLWILF

IVTIAGNATVLFSTWRRKRKSRMTYFVTQLAITDSFTGLINIMTDIIWRYTGDFMAPDIV

CRVVRYFQVVLLYASTYVLVSLSIDRYHAIVYPMKFLQGERQAKVLIGVAWSLSFLFSIP

TLIIFGKRQLSNGEVQCWALWPDESYWIPYMTVVAFLVYFIPLIIISVIYSIVIRTIWMK

SKAQAAIISSCPGGKTSRGYTSRGFISRAKVKAIKYSIVIILAFILCWSPYFLFDILDNF

NILPETKERFYASVIIQNLPALNSAVNPLIYCFFSNSLCPPSEERRTQRLGGTFRERSDG

GQQMQVLSKPECI

>opossum NPSR

MQVNGTEGSWAVNGTMEPLDAQPLDTCAETVTSPEGAEQEEWGSFYYSFKTEQLVTLWILFVFTIVGNAFVLFSTWKRKRKSRMTFFVTQLAITDSFTGLINILTDIIWRFTGDFMAPDLICRVIRYLQVVLLYASTYVLVSLSIDRYHAIVYPMKFLQGEKQAKVLIVVAWSLSFLFSIPTLIIFGKRKLANGEVQCWALWPDDSYWIPYMTIVAFLVYFIPLIIISVIYFIVIRTIWIKSKAHDMIISNCSEGKLCSSYNRGVISKAKIKAIKYSIVIIFAFVICWSPYFLFDILDNFSLLPETKERFYASVIIQNLPALNSAINPLIYCLFSNSLCYPCRERRNAGGTFQDKTERHEMRMRPKPEFI

>cow NPSR MPANLTEGSAKSNGTGQALNSPVLCTETVTFTAEVEGEEWGSFYYSFKTEQLITLWVLFVFTIVGNAIVLFSTWRRKRKSRMTFFVTQLAITDSFTGLVNILTDIIWRFTGDFMAPDLVCRVVRYLQVVLLYASTYVLVSLSIDRYHAIVYPMKFLQGEKQAKVLIMIAWSLSFLFSIPTLIIFGKRKLSNGEVQCWALWPDDSYWTPYMTIVAFLVYFIPLTIISVIYAIVIRTIWVKSKAYETVISNCSDGKLCTSYNRGLISKAKIKAIKYSIVIILAFICCWSPYFLFDILDNFSLLPNTKERFYASVIIQNLPALNSAINPLIYCVFSNSICFPCREQRSRDSRMTCRERTEKHEMQVLSKPEFM

>dog NPSR

MPAHLAEASPDSSHPSGATQTLEASPVLCTEVVTFTQVVEREEWGSFYYSFKTEQLITLWVLFVFTVVGNSMVLFSTWRRKRKSRMTFFVTQLAITDSFTGLVNILTDIIWRFTGDFMAPDLVCRVVRYLQVVLLYASTYVLVSLSIDRYHAIVYPMKFLQGEKQAKVLVMIAWSLSFLFSIPTLIIFGKRKLPNGEVQCWALWPDDSYWTPYMTIVAFLVYFIPLTIISVIYAIVIRTIWIKSKAHETVISNCSGGKLCTSYNRGLISKAKIKAIKYSIVIILAFICCWSPYFLFDILDNFSLLPDTEERFYASVIIQNLPALNSAINPLIYCVFSSSICFPCREQTSRGSRKTFRERTQRHEMQVLSKPEFI

>giant panda NPSR

MPANLTEGSPDSNDASGTTQTLDASPVLCTETVTFTEVVEGEQWGSFYYSFKTEQLITLWVLFVFTIVGNSMVLFSTWRRKRKSRMTFFVTQLAITDSFTGLINILTDIIWRFTGDFLAPDLVCRVVRYLQVVLLYASTYVLVSLSIDRYHAIVYPMKFLQGEKQAKVLIVIAWSLSFLFSIPTLIIFGKRKLSNGEVQCWALWPDDSYWTPYMTIVAFLVYFIPLTIISVIYAIVIRTIWIKSKAHETVISNCLDGKLCTSYHRGLISKAKIKAIKYSIVIILAFICCWSPYFFFDILDNFRLLPDTEERFYASVIIQNLPALNSAVNPLIYCVFSSSVCFPCRERTSRDSRMTFRERTQRHEMQVLPKPEFI

>orangutan NPSR

MPANFTEGSFDSSGTGQTLDSSPVACTETVTFTEVVEGKEMGLPFYYSFKTEQLITLWVLFVFTIVGNSVVLFSTWRRKKKSRMTFFVTQLAITDSFTGLVNILTDIIWRFTGDFMAPDLVCRVVRYLQVVLLYASTYVLVSLSIDRYHAIVYPMKFLQGEKQARVLIVIAWTLSFLFSVPTLIIFGKRTLSNGEVQCWALWPDDSYWTPYMTIVAFLVYFIPLTIISIMYGIVIRTIWIKSKTYETVISNCSDGKLCSSYNRGLISKAKIKAIKYSIIIILAFICCWSPYFLFDILDNFNLLPDTQERFHASVIIQNLPALNSAINPLIYCVFSSSISFPCRERRSQDSRMTFRERTERHEMQILSKPEFI

>frog NPSR

MQVSFAEASDFSNTSSGTLNLTLTNCTSANSSADIEEEYAWESFYTSFKMEQLITLWILFIVTIFGNSVVLYLTWKERKRKSRMTFFVTQLAITDYLTGIISISVNIIWRFTGEFMAPEIVCKTVRYLQVVLLYASTYVLVSLSIDRYHAIVHPMKFLQGEKQAKVLIAVSWTLSFLFSIPTFIIFGKLKLPNGEMQCWALWPDDSYWTPYMTIVALLVYFIPLIIISVIYFIVIRTIWVKSKGHAVIISNYTDGNFCTSYSHRGLISKAKMKAIKYSIVVILAFILCWSPYFLFDILDNFEMLPETKERFYASVIIQHLPFLNSAINPIIYCVFSNRHCRLSRDRNSGKLGGTIRDKTEGIEMQVVSRPEYL

>rabbit NPSR

MPANHTEGSAPSTQSTLTQGSSPATCTETETFTEVVEGEEWGSFYYSFKTEQLVTLWVLFVFTIVGNSVVLFSLWRRKRKSRMTFFVTQLAITDSFTGLVNILTDIIWRFTGDFMAPDLVCRVVRYLQVVLLYASTYVLVSLSIDRYHAIVHPMKFLQGEKQAKVLIGIAWSLSFLFSIPTLIIFGKRTLSNGEVQCWALWPDDSYWTPYMTIVAFLVYFIPLTIISVIYGIVIRTIWVKSKACETVNSNCSDGKLCTSYNRGLISKAKIKAVKYSAAIILAFICCWSPYFLFDILDNFNFLPNTKERFYASVIIQNLPALNSAINPLIYCVFSSTVCFPFRERRSQDSRMTCRERTERHEMQTLAKPEFL

>gorilla NPSR

MPANFTEGSFDSSGTGQTLDSSPVACTETVTFTEVVEGKEWGSFYYSFKTEQLITLWVLF

VFTIVGNSVVLFSTWRRKKKSRMTFFVTQLAITDSFTGLVNILTDIIWRFTGDFMAPDLV

CRVVRYLQVVLLYASTYVLVSLSIDRYHAIVYPMKFLQGEKQARVLIVIAWSLSFLFSIP

TLIIFGKRTLSNGEVQCWALWPDDSYWTPYMTIVAFLVYFIPLTIISIMYGIVIRTIWIK

SKTYETVISNCSDGKLCSSYNRGLISKAKIKAIKYSIIIILAFICCWSPYFLFDILDNFN

LLPDTQERFYASVIIQNLPALNSAINPLIYCVFSSSISFPCRANSSVYLLACDVSVLWAL

VPTSEKESCESWRRKGAKITGFQNDVPGENREA

>elephant NPSR

MPANLTEGSPDSSGTTQMLDSSPAACSETVTVTEVVEGEEWGSFYSSFKTEQLITLWVLF

VFTTVGNAIVLFSTWRRKRKSRMTFFVTQLAITDSFTGLFNILTDIIWRFTGDFMAPDLV

CRVVRYFQVVLLYASTYVLVSLSIDRYHAIVYPMKFLQGEKQAKVLIVIAWSLSFLFSIP

TLIIFGKRKLSNGEVQCWALWPDDSYWTPYMTIVAFLVYFIPLTIISVIYGIVIRTIWIK

SKAHDMVISNCSNGKLCTSYNRRLISKAKIKAIKYSVVIILAFICCWSPYFLFDILDNFS

ILPDTKERFYASVIIQNLPALNSAINPLIYCAFSSSICFPCRERKSRDSRMTCQERTERH

EMQVLSKPEFI

>lancelet NPSR-like*^+^

NYLVTLQTEQLVTLWVLFVFIVVGNSLVLLIMWLERNKTSRMNFFILNLAVADLSAGLFNVLPDIVHRFVVEWIAGNTLCKLVKYTQAVLLYASTYVLVAMSIDRYDAIVHPLQFVREHKSKVMISVAWGLALLFSVPSPVIFAVRRQPNGEWQCWAEWPEDWYWVPYMTVVAAFVFFIPLGIISICYIAIIVKIWKRGKGMAYEDHIPRSRASSGKNFYRIFHLIHINNGFTSRAKARTIKLSVAIILAFICCWSPYFLFDILDNYDALPDTQAKKEASLIIQNLPALNSAINPIIYGFFSTKLYRKLR

>acorn worm NPSR-like^+^

MTSKLNEMKYAAVMGAFINTTVYNNTVNNSTYDIPEKHQSVLSMYQTEQLILLWFLFAFVVIGNAIVLVSVCLVRHKKSRMNFFIMNLAIADLSVGLLNILPDIIHRYTREFYGGEIVCKLVKYVQAIVVYGSTYQLVALSIDRYDAIVHPMNFSGNKRSMIMVISMWVVAFILAVPSPVFFEETVLENGEVQCWIELPQTWVWKPYSVILAFLLFFIPLVIVTFCYSVIIYTIWRKSKMMVPAKRRIFNEKNGDSRGLIPKAKIKTIKMTLCIVLSFIVCWSPFTLWFILEIYGHIPKNDLTMTIHIIVQNLPSLNSATNPAIYGLFSTNICKELR

>pig NPSR

MPASLTEDSPVTNGTEQAPDSPVLCTATVTFTAVAEGEEWGSFYYSFKTEQLITLWVLFVFTIVGNAVVLFSTWRRKRKSRMTFFIAXLALSDSFTGLVNILTDIIWRFTGDFMAPDLVCRVVRYLQVVLLYASTYVLVSLSIDRYHAIVYPMKFLQGEKQAKILIAIAWSLSFLFSIPTLIIFGKRELSNGEVQCWALWPDDSYWTPYMTIVAFLVYFIPLTIISIIYAIVIRTIWIKSKAHETVISNCSDGKLCTSYNRGLISKAKIKAIKYSIVIILAFICCWSPYFLFDILDNFSLLPNTKDRFYASVIIQNLPALNSAINPLIYCVFSSSICFPCGEQRSRDSRMTCRERIERHEMQVLSKPEFM

>turkey NPSR

MEVGPTAASSSSPAGTGWLDVTFVTCTDTVTFTEVLEEEEWKSFYYSFKTEQLVTLWILFIVTIAGNAIVLFSTWRRKRKSRMTFFVTQLAITDSFTGLINIMTDIVWRYTGDFMAPDIVCRVVRYLQVVLLYASTYVLVSLSIDRYHAIVYPMKFMQGERQAKVLIGVAWSLSFLFSIPTLIIFGKRQLSNGEVQCWALWPDDSYWIPYMTVVAFLVYFIPLIIISVIYSIVIRTIWMKSKAQAVIISSCTDGRTSAGYTSRGFISRAKVKAIKYSIVIVLAFALCWSPYFLFDILDNFNILPETKERFYASVIIQNLPALNSAINPLIYCLFSNHLCTPFEERRTRRLEGTFRDRSDGGQEMQVLSKPEYI

>alpaca NPSR °

SFTGLVNILTDIIWRFTGDFMAPDLVCRVVRYLQXXXXXXXXXXXXXXXXXXXXXXXXXXXXXXXXKQAKVLIVIAWSLSFLFSIPTLIIFGKRKLSNGEVQCWALWPDDSYWTPYMTIVAFLVYFIPLTIIXXXXXXXXXXXXXXXXXXXXXXXXXXXGKLCTSYSRGLISKAKIKAIKYSIVIILAFICCWSPYFLFDILDNFSLLPNTKERFYASVIIQNLPALNSAINPLIYCVFSGSICFPCGANSSSDLLASDISVLWALVLTSGKEGCEQGRKGAKITGFQNDMSGEK

>armadillo NPSR °

MPASLTERSPNSNRRTQTPDYPPVTCTETVTFTEVVEGEEWGSFYYSFNXXXXXXXXXXXXXXXXXXXXXXXXXXXXXXXXXXXXXXXXXXXXXSFTGLVNILTDIIWRFTGDFLAPDLVCRVVRYLQVVLLYASTYVLVSLSIDRYHAIVYPMKFLQGXXXXXXXXXXXXXXXXXXXXXXXXXXXXXXXXXXXXXXXXXXXXXXXXXXXXXXXXXXXXXXXXXXXXIIYGIVIRTIWIKSKAHETVISNCSGGKLCTSYNRGLISKAKIKAIKYSVVIVLAFICCWSPYFLFDILDNFSLLPDTSERFYASVIIQNLPALNSAINPLIYCVFSSSICFPGRVISKAFFLASDILVHWVLMLTSGEEGREWWGRKGAKITGFQNDWPREN

>cat NPSR °

MPANLTEGSPDSKGSSGTTQTPDASPALCTETVTFTEVVEGEEWGSFYYSFKXXXXXXXXXXXXXXXXXXXXXXXXXXXXXXXXXXXXXXXXXXXXXXFTGLNITDIWRFTGDFMADLVCRVVRYLQXXXXXXXXXXXXXXXXXXXXXXXXXXXXXXXXKQAKVLIMIAWTLSFLFSIPTLIIFGKRKLSNGEVQCWALWPDDSYWTPYMTIVAFLVYFIPLTIIXXXXXXXXXXXXXXXXXXXXXXXXXXXGKLCTSYNRGLISKAKIKAIKYSIVIILAFICCWSPYFLFDMLDNFSLLPDTEERFYASVIIQNLPALNSAINPLIYCVFSSSICFPYGTRSSAYLLASDVSVHWALVLTSGKWEGRKAANITGQ

>hedgehog NPSR °

MSASLTEGSANSKATMQTLDSSSVLCTQVVTFTKVVEEEWSSFYNSFKTEQLITLWVLFVFTIVGNATVLLSTWRRKRKSRMTFFVTQLAITDSFTGLVNILTDIIWRFTGDFLAPDLVCRVVRYFQXXXXXXXXXXXXXXXIDRYHAIVYLMKFLQRXXXXXXXXXXXXXXXXXXXXXXXXXXXXXXXXXXXXXXXXXXXXXXXXXXXXXXXXXXXXXXXXXXXXVIYAIIIRTIWIKSKAHETVISNCSDGRLCTSYNRRLISKAKIKAIKYSIVIIFGFIFCWSPYFLFDILDNFSLLPDTKERFYASVIIQNLPALNSAINPLIYCMFSHSLCLLCKVISSVCHLVRDVSVHWTLIPPCKEVFKWRRKGAQITGFKSDLSGEKQ

>hyrax NPSR °

MPANLTEGSSNSSRTIQMWDSSPVACTDTVTVTEVVEGEEWGSFYHSFKTEQLITLWVLFVFTTVGNAFVLFSTWRRKRKSRMTFFVTQLAITDSFTGLVNILTDIIWRFTGDFMAPDLVCRVVRYLQVVLLYASTYVLVSLSIDRYHAIVYPMKFLQGEKQAKILIVIAWSLSFLFSIPTLIIFGKRKLPNGEVQCWALWPDDSYWTPYMTIVAFLVYFIPLTIISTIYGIVIRTIWIKSKARETVISNCSXXXXXXXXXXXXXXXXXXXXXXXXXXXXXXFICCWSPYFLFDILDNFNVLPHTKERFYASVIIQNLPALNSAINPLIYCVFSSSTCFPCGSNNSSHFLASKGSACVLVLTSKEEVWKQRRKGAKITGFQNDLPGGN

>kangaroo rat NPSR °

MPTSLPEGSSLSTWTVPGPDPSLVACTGTVAFTEEVEHEEGGSIYSSFRTEQLLTLWVLFIITIVGNSVVLYSTWRRKRKSRMTFFVTQLAITXXXXXXXXXXXXXXXXXXXXXXXXXXXXXXXXXXXXXXXXXXXXXXXXXXXXXXXXXXXXXXXXXXXXXXXXXXXXXXXXXXXXXXXXXXXXXXXXXXXXXXXXXXXXXXXXXXXXXXXXXXXXXXXXXXXXXXVIYGIVIRTIWIKSKAQEMVISNCSDGKLCTSYSRGVISKAKIKAIKYSIVIILAFICCWSPYFLFDILDNFNLLPDTKERFFASVIIQNLPALNSAINPLIYCAFSGSPCGPCRVNRSVQDFVVVAVHTLVLTNGNGNEREGSKVTRFQNNMPREKR

>lesser hedgehog tenrec NPSR °

TEQLVTLWVLFVFTIVGNATVLFSTWRRKRKSRMTFFVTQLAITDSFTGLVNILTDIIWRFTGDFMAPDLVCRVVRYLQVVLLYASTYVLVSLSIDRYHAIVYPMKFLQGEKQAKVLVIAWALSFLFSIPTLIIFGKRKLSNGEVQCWAVWPDDSYWTPYMTIVAVLVYFIPLTIISVIYGIVIRTIWIKSKAHETVISNCSXXXXXXXXXXXXXXXXXXXXXXXXXXXXXXFICCWSYFLFDMLDNFSVLPDTRERFYASVIIQNLPALNSAINPLIYCVFSSSICSPYRANNSSYILASDGSVHRLLVLTSGKERCKGRKGTEITGFQNDLPREN

>microbat NPSR °

TEQLITLWVLFVFTIVGNSAVLFSTWRRKRKSRMTFFVTQLAITXXXXXXXXXXXXXXXXXXXXXXXXXXXXXXXXXXXVVLLYASSYVLVSLSIDRYHAIVYPMKFLQGEKQAKVLIATAWGLSFLFAIPTLIIFGKRKLSNGEVQCWALWPDDSYWTPYMTIVAFLVYFIPLAIISVIYTIVIRTIWIKSKAQEMVISNCSDGKLCTRYNRGLFSKAKIKAIKYSMVIILXXXXXXXXXXXXXXXXXXXXXXXXXXXXXXXXXXXXXXXXXXXXXXXXXXXXXXXXXXXXXANNSAYLLARDVCSWALVLAGKEGCDWWGREGAEITGFQNHLSGEG

>pika NPSR °

MPANWTEGSTTSNQTTLTLGLSPVTCTATVTFTEVVDQEEWGSFYYSFKTEQLITLWVLFVFTIVGNSVVLFSTRRRKRXXXXXXXXXXXXXXXSFTGLVILTDIIWRFTGDFMAPDLICRVVRYLQVVLLYASTYVLVSLSIDRYHAIVHPMKFLQGEKQAKVLIGIAWGLSFLFSIPTLIIFGKRTLSNGEVQCWALWPDDSYWTPYMTIVALLVYFIPLTIISVIYGIVIRTIWVKSKAYDTVISNCSDGKLCTSYNRGLISKAKIKAIKYSVVIILAFICCWSPYFLFDILDNFNLLPDTKERFYASVIIQNLPALNSAINPLIYCVFSSSICLPFGAHSSILLIDDPDLWALMKVKEHYEWKRNKAKITGFQNDLPGQN

>shrew NPSR °

MPVNRTEDTPTSNPTWETPDPSPGLCTQTVTFTEETERAEWGSFYYSFKTEQLITLWVLFIFTIVGNSTVLLSTWRRKRKSRMTFFVTQLAITDSFTGLVNILTDIIWRFTGDFMAPDLVCRVVRYLQVVLLYASTYVLVSLSIDRYHAIVYPMKFLQGEKQAKVLIVIAWSLSFLFSIPTLIIFGKRKLSNGEVQCWALWPDDSYWTPYMTIVAFLVYFIPLTIIXXXXXXXXXXXXXXXXXXXXXXXXXXXVKLCTSHNRGLISKARTKAIKYSAVIILAFICCWSPYFLFDILDNFNLLPDTKERFYASVIIQNLPALNSAINPLIYCVFNGSVCFPCRASSCSLQDFPVHWTLVLTSGKEACGRQGAKIARFQNDTPGES

>sloth NPSR °

MPANLTEGSPNPNANTQTVDYSPATCTDTVTFTEVVEGEDWGSFYYSFKXXXXXXXXXXXXXXXXXXXXXXXXXXXXXXXXXXXXXVTQLVITDSTGLVSILTDIIWRFTGDFLAPDPVCRVVRYLQVVLLYASTYVLVSLSIDRYHAIVYPMKFLQGEKQAKVLIVIAWSLSFLFSIPTLIIFGKRKLSNGEVQCWALWPDDSYWTPYMTIVAFLVYFIPLTIISIIY

>squirrel NPSR °

TEQLITLWVLFVFTIVGNAVVLFSTWKRKRKSRMTFFVTQLAITXXXXXXXXXXXXXXXXXXXXXXXXXXXXXXXXXXXVVLLYASTYVLVSLSIDRYHAIVYPMKFLQGEKQAKVLIGVAWSLSFLFSIPTLIIFGKRTLSNGEVQCWALWPDDSYWTPYMTIVAFLVYFIPLTIISVIYGIVIRTIWIKSKAQEMVISNCSDGKLCTSYNRGLISKAKIKAIKYSIVIILXXXXXXXXXXXXXXXXXXXXXXXXXXXXXXXXXXXXLPALNSAINPLIYCIFSGSICYPCRANSGVFLANSVSVHWGLVLTVCKWWRGKGAKITRLNDMPRKNG

>tarsier NPSR °

MSVFTDDSXXXXXXXXXXXXXXXXXXXXXXXXXXVEREWGSFYYSFKXXXXXXXXXXXXXXXXXXXXXXVLPWRRKRKSRMTFFVTQLAITXXXXXXXXXXXXXXXXXXXXXXXXXXXXXXXXXXXVVLLYASTYVLVSLSIDRYHAIVHPMKFLQGEKQAKVLIMIAWSLSFLFSIPTLVIFGKRTLSNGEVQCWALWPDDSYWTPYMTIVAFLVYFIPLTIIXXXXXXXXXXXXXXXXXXXXXXXXXXXGKLCSSYNRRLISKAKMKAIKYSIIIILAFICCWSPYFFFDILDNFNLLPDTKERFYASVIIQNLPALNSAINPLIYCVFSNSICFPC

>wallaby NPSR °

MQVNVTEGSWTVNGTMEPQDSSPLASCAETVTFPEVVEEEEWGSFYYSFKTEQLVTLWVLFVFTVVGNAFVLFSTWKKKRKSRMTFFVTQLAITXXXXXXXXXXXXXXXXXXXXXXXXXXXXXXXXXXXVVLLYASTYVLVSLSIDRYHAIVHPLKILQGEKQAKVLIFIAWSLSFLFSIPTLIIFGKRKLANGEIQCWALWPDDSYWIPYMTIVAFLVYFIPLIIISVIYFIVIRTIWIKSKAHDRIISDCSXXXXXXXXXXXXXXXXXXXXXXXXXXXXXXFVICWSPYFLFDILDNFSLLPKTKERFYASVIIQNLPALNSAINPLIYCLFSNSLCYPCGKKGCTRDFPGENR

**Note:**

Sequences corrected manually at N and C- termini are represented with * and ^+^ at the end of the sequence names

Fragmented/ partial sequences not used for further analysis are indicated with ° symbol at the end of the sequence names.
